# MARIA (Medical Assistance and Rehabilitation Intelligent Agent) for Medication Adherence in Patients With Heart Failure: Empirical Results From a Wizard of Oz Systematic Conversational Agent Design Clinical Protocol

**DOI:** 10.2196/55846

**Published:** 2025-04-10

**Authors:** Nik Nailah Abdullah, Jia Tang, Hemad Fetrati, Nor Fadhilah Binti Kaukiah, Sahrin Bin Saharudin, Vee Sim Yong, Chia How Yen

**Affiliations:** 1 School of Information Technology Monash University Malaysia Petaling Jaya Malaysia; 2 Faculty of Information Technology Monash University Melbourne Australia; 3 Faculty of Computer Science Dalhousie University Halifax, NS Canada; 4 Clinical Research Center (Hospital Queen Elizabeth II) Institute for Clinical Research National Institute of Health Kota Kinabalu Malaysia; 5 Sabah Scientific Research Society Kota Kinabalu Malaysia

**Keywords:** heart failure, medication adherence, self-monitoring, chatbot, conversational agent, Wizard of Oz, digital health

## Abstract

**Background:**

Nonadherence to medication is a key factor contributing to high heart failure (HF) rehospitalization rates. A conversational agent (CA) or chatbot is a technology that can enhance medication adherence by helping patients self-manage their medication routines at home.

**Objective:**

This study outlines the conception of a design method for developing a CA to support patients in medication adherence, utilizing design thinking as the primary process for gathering requirements, prototyping, and testing. We apply this design method to the ongoing development of Medical Assistance and Rehabilitation Intelligent Agent (MARIA), a rule-based CA.

**Methods:**

Following the design thinking process, at the ideation stage, we engaged a multidisciplinary group of stakeholders (patients and pharmacists) to elicit requirements for the early conception of MARIA. In collaboration with pharmacists, we structured MARIA’s dialogue into a workflow based on Adlerian therapy, a psychoeducational theory. At the testing stage, we conducted an observational study using the Wizard of Oz (WoZ) research method to simulate the MARIA prototype with 20 patient participants. This approach validated and refined our application of Adlerian therapy in the CA’s dialogue. We incorporated human-likeness and trust scoring into user satisfaction assessments after each WoZ session to evaluate MARIA’s feasibility and acceptance of medication adherence. Dialogue data collected through WoZ simulations were analyzed using a coding analysis technique.

**Results:**

Our design method for the CA revealed gaps in MARIA’s conception, including (1) handling negative responses, (2) appropriate use of emoticons to enhance human-likeness, (3) system feedback mechanisms during turn-taking delays, and (4) defining the extent to which a CA can communicate on behalf of a health care provider regarding medication adherence.

**Conclusions:**

The design thinking process provided interactive steps to involve users early in the development of a CA. Notably, the use of WoZ in an observational clinical protocol highlighted the following: (1) coding analysis offered guidelines for modeling CA dialogue with patient safety in mind; (2) incorporating human-likeness and trust in user satisfaction assessments provided insights into attributes that foster patient trust in a CA; and (3) the application of Adlerian therapy demonstrated its effectiveness in motivating patients with HF to adhere to medication within a CA framework. In conclusion, our method is valuable for modeling and validating CA interactions with patients, assessing system reliability, user expectations, and constraints. It can guide designers in leveraging existing CA technologies, such as ChatGPT or AWS Lex, for adaptation in health care settings.

## Introduction

### Background and Motivation

Heart failure (HF) is a global concern associated with significant morbidity and mortality [1]. Recent findings from the ASIAN‐HF registry suggest a potential shift in the HF burden from North America, Western Europe, and Eastern Europe to the Asia-Pacific region [2].

According to the ASIAN‐HF registry, within Asia, Southeast Asian patients have the highest burden of risk factors and worse outcomes than Northeast and South Asian patients [2,3]. This burden pressures individuals, their families, and the health care systems through various costs, with the most prominent being repeated hospitalizations [1]. For example, as high as 10% of hospital admissions are related to HF. The total HF costs accounted for approximately 1.8% of total health expenditure [4].

Studies show that HF’s rehospitalization and mortality rates were influenced by patients’ medication nonadherence [5-7]. As poor self-motivation and inadequate medication knowledge are the typical reasons for medication nonadherence, doctors and health care workers should emphasize the importance of medication adherence by constantly providing appropriate encouragement and education to patients [8,9].

Research has shown that some of these factors leading to hospitalizations are preventable by close home monitoring supported by family or nurse practitioners [6]. Nonetheless, such programs are challenging to apply in our local setting due to the limited number of specialized HF nurses who can support the wider HF patient population.

Therefore, we explore related work that uses conversational agent (CA), a type of artificial intelligence (AI) application that can be leveraged to assist in the self-monitoring of patients with HF in the following section.

A CA is a computer program capable of understanding natural human language (in text, speech, or both forms) and responding autonomously using the same language [10]. They can be accessed through a variety of ways, such as social media platforms (eg, Facebook Messenger), websites, and smartphone apps, or deployed using stand-alone digital devices (eg, Alexa, Google Assistant, and Siri). The first CA, ELIZA, was created by Joseph Weizenbaum at the Massachusetts Institute of Technology in 1966 [11].

ELIZA was developed to converse with the users via text, imitating a psychotherapist, to fool them into believing that they were talking to a human being. Today, thanks to technological advancements in AI, CAs can handle much more complex tasks in a wide variety of fields, including finance, education, travel, and retail [12-15], and they are predicted to be used even more widely in the future [16].

**Figure 1:**
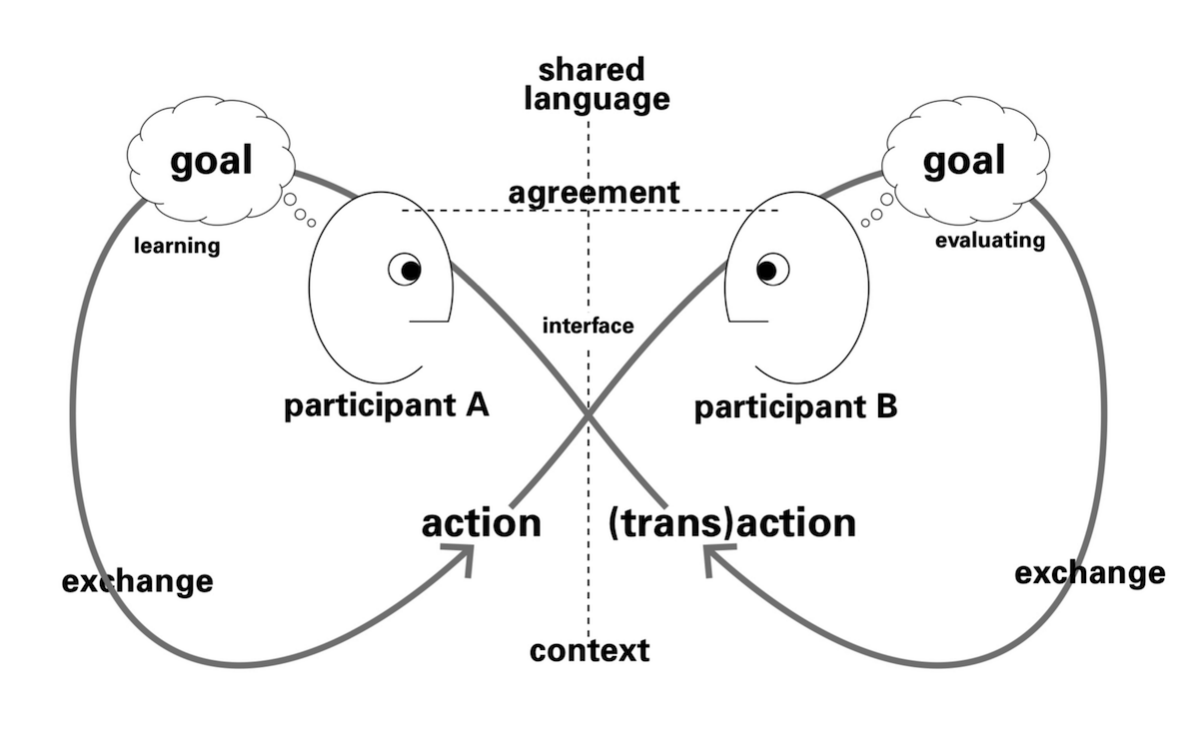
Simplified view of conversation theory.

Engaging in natural conversation with humans is the main characteristic of CA, and current methods refer to conversation theory (demonstrated in Figure 1 [17]), such as using advanced machine learning methods to extract users’ intents from their utterances (speech) [18].

For a CA to produce natural conversations in a narrative manner, the format of the content must be outlined through rule-based workflows, templates, or intent-driven approaches to create an output. Every CA that uses a natural language system relies on narrative design, also called conversation design, to produce that output.

Conversational design combines several disciplines, including copywriting, user experience design, interaction design, visual design, motion design, and, if relevant, voice and audio design. Conversation design not only requires using natural conversational language but also creates logically sound conversational flow and design specifications that capture the entire user experience. More recently, machine learning capabilities have been used in CA to provide the ability to learn from the data so that an adaptable context of responses can be provided to the users.

There are several ways to generate the responses. First, is the rule-based method in which the CA produces a response by selecting it from a pool of predetermined responses either following simple rules to match phrases or identifying specific keywords in the text [19].

The second type is the generative-based CAs, which use AI algorithms to develop a contextual response informed by the system’s previous and ongoing learning [20].

Rule-based CAs allow developers greater control over the conversation content and flow, which is a useful feature when developing CAs for health care. By contrast, AI algorithms, particularly neural networks, may develop decisions that are not explainable or understood by the end user, referred to as the *black box* [20]. In health care settings, the *black box* effect may lead to biased or erroneous decision-making and patient harm which is highly dependent on the type of algorithms used to learn and generate the responses.

Therefore, in our work, we choose to develop a rule-based CA, given that it will allow developers better control and transparency in the responses.

Researchers have effectively innovated the application of CA in the digital health (DH) area, covering functions such as scheduling doctor appointments, monitoring medication intake, checking symptoms, diagnosing, providing treatment plans, and helping patients with rehabilitation [21-24]. DH has a broad scope that includes categories such as mobile health, health information technology, wearable devices, telehealth and telemedicine, and personalized medicine [7].

There are existing applications developed for supporting patients with HF. CARDIAC is a human-centered conversational assistant that helps patients with HF monitor their health status through reminders, question answering, relevant data collection, and generating data tendencies and personal health records [25]. Another CA, DIL, improves the self-care and quality of life of patients with HF by motivating them to adhere to a healthy lifestyle, including a controlled diet, a continuous medication routine, and regular exercise [26]. As a medication advisor, CARMIE speaks in Portuguese and interacts with patients with HF in real time to provide quality answers to medication-related questions according to its knowledge representation model and patients’ prescriptions [27].

Based on our literature review [10-12,26,28-31], the existing CAs in the HF area concentrated on developing functional features’ effectiveness and accuracy. However, no study has specifically displayed a method for building agents’ natural language–based conversations to encourage and educate patients with HF about medication adherence, nor a standard for evaluating this type of CA design early in the development stage as a DH solution.

Therefore, our study aims to adopt established design methods and conceive them into a systematic method that uses a clinical observational study protocol. We use observational study protocol to produce new knowledge in improving conversational design, examine acceptability, and reduce uncertainties in the harmful effects of using CA in medication adherence. It will fill the gap of the existing studies in the DH domain in designing a CA (or chatbot) that encourages and educates patients about medication adherence.

### Prior Work

#### Overview

In the following subsections, we will review the prior work in related research studies.

#### Designing a CA Agent With Human-Likeness Attributes

To fill the gap in the existing studies and strategically motivate patients to change medication adherence behavior, we searched for suitable psychological theories to support our CA dialogue. Adlerian psychoeducational therapy emphasizes that encouragement is the key to achieving an individual’s growth and development [13]. Developed by Alfred Adler [32], the approach states that the motivation of an individual’s behavior change can be goal oriented and related to one’s relationship with others and contributions to society [14]. This therapy aims to help individuals identify their mistaken beliefs in their capabilities and apply appropriate improvements to reinforce their strengths and compensate for their weaknesses. It encourages individuals to regain their confidence in achieving their goals. The therapy is widely used in mental health treatment for anxiety, depression, behavior disorders, mental disorders, and career encouragement [15]. Adlerian psychologists encourage their patients by using therapeutic skills. For instance, they enhance patients’ self-efficacy and affirm patients’ capabilities and potentials by narrating other patients’ successful experiences to build good examples. They help patients recognize and believe in their strengths, resources, progress, and positive sides of life experiences and encourage them to keep striving toward their goals [16].

The storytelling method to encourage individuals to learn how relevant peers have successfully solved a similar problem is also conceptualized in Social Cognitive Theory [33,34]. Being expanded by Albert Bandura [35], Social Cognitive Theory studies individuals’ behavior change through the impact of individuals’ experiences, the achievements of others, and the influences from surroundings [36]. The theory believes that an individual could learn similar behaviors from observing the successful experiences of others [37].

The Tripartite Encouragement Model is a psychological framework that combines the insights of encouragement, verbal persuasion, and character strength and virtues [16]. The Tripartite Encouragement Model introduces the concept of effective encouragement to optimize the positive influences of encouragement to recipients. An encouragement message could effectively motivate recipients’ self-efficacy by emphasizing their progress rather than pointing out their distance apart from the target. Highlighting the process-oriented factors is another way to improve the effectiveness of encouragement, such as emphasizing the recipient’s positive effort, attitude, and feelings.

Cialdini and Sagarin’s [18] principles of interpersonal influence contain psychological persuasion strategies to trigger individuals’ acceptance of requests while hesitating. The principle of commitment and consistency states that individuals tend to accept a request consistent with their committed position [18]. The 4-wall technique asks individuals several easy-to-say-“yes” questions first, then leads them to comply with the final crucial request [38]. The principle of reciprocity demonstrates that individuals tend to accept a request if requestors offer a concession [18]. The reciprocal concession procedure significantly reduces the requested content after the initial request gets rejected, which could make the new request more acceptable [39].

Anthropomorphism, or human-likeness, is a phenomenon that also occurs in human-technology interaction contexts. It is used to enhance user experience in chatbots. This approach is typically implemented through the CA or chatbot’s visual representation, such as an illustration, image, or animated avatar, alongside a persona that defines various humanlike characteristics, including sex, gender, education, race, and age [40,41]. These features are often selected to reflect the target audience, such as an avatar having a similar skin tone, wearing local attire, or having a common local name [42]. Additionally, conversation style plays a crucial role, with the use of slang, local accents, and culturally appropriate vocabulary tailored to the users’ demographic [40]. Another significant factor in shaping a chatbot’s humanlike persona is its social role. For example, adopting a peer persona or an expert persona (eg, a doctor) has been shown to be effective, particularly in medical-related chatbots [40].

The existing design guidelines for CAs explain that similarity attraction significantly impacts users’ acceptance of the system because individuals tend to apply human-human interaction to engage with virtual agents [43]. Individuals prefer to engage with those with similar experiences or interests, and the similarities could create more conversations to establish relationships and trust [44]. Existing studies also suggest that the human-likeness of the CA is essential [43]. Human beings spontaneously mix emotions and languages to display their feelings and reactions during face-to-face conversations. Emojis can display speakers’ emotions and optimize the chatting experiences during text-based online communication [45]. Some studies recommend adding an intentional pause between messages sent and received to generate a natural feeling as chatting with a human [46]. The pause will also allow users to think and type their responses [47]. When applying encouragement and education strategies, the credibility appeal could be enhanced by providing reliable evidence of the information to users [48]. Furthermore, people tend to trust an individual with a consistent personality that indicates one’s capability, predictability, and reliability [43]. The patterns in language use could reveal one’s personality [49]. Moreover, finding the right balance of anthropomorphism—without overdoing it, which can diminish the sense of human-likeness—has been shown to increase user engagement, compliance, satisfaction, and the intention to reuse chatbots [50].

In applying an agent-based concept in modeling CA, protocols play a central role in agent communication with humans or another CA. A protocol specifies the rules of interaction between 2 or more communicating agents by restricting the range of allowed follow-up utterances for each agent at any stage during a communicative interaction (dialogue). Such a protocol may be imposed by the designer of a particular system or it may have been agreed upon by the agents taking part in a particular communicative interaction before that interaction takes place [51].

#### Wizard of Oz Procedure in the Elicitation of Requirements and User Experience

Wizard of Oz (WoZ) is a well-established method for simulating the functionality and user experience of future systems, where humans simulate all or part of the behaviors and functionalities of an automated system [52,53]. Using a human wizard to mimic certain operations of a potential system is particularly useful in situations where extensive engineering effort would otherwise be needed to explore the design possibilities offered by such operations [53].

The term “Wizard of Oz (WoZ)” was first coined by John Kelley [54], who used this technique to simulate a calendar application that could be operated via natural language input [53]. The method was also occasionally referred to as “Pay No Attention to the Man Behind the Curtain” and “OZ paradigm” [53,55]. Over time, the use of WoZ expanded beyond the use of simulating text-based interfaces to include interfaces involving speech, gesture, facial recognition, and multimodal user interactions [53,56-58].

There are several key uses of the WoZ method for designing interactive systems. One major application is in interaction design, where WoZ is used to explore human-computer dialogues and interaction strategies. Additionally, WoZ is used to collect text and speech corpora (ie, eliciting requirements), which aids both interaction design and engineering work by training and fine-tuning technology components. A third key use involves employing WoZ to develop early prototype technology components, allowing for the evaluation of system performance in specific application areas without the need for full-scale engineering efforts. Overall, these uses fall into 4 broad categories: exploring interaction strategies, designing dialogues, collecting corpora, and evaluating system components [53].

In recent years, researchers have utilized WoZ for various purposes within these categories, such as building a data set to create a virtual assistant for helping programmers use application programming interfaces [59], simulating autonomous driving cars [60,61], developing drive-assist features [62], conducting virtual reality elicitation studies [63], and creating a mixed reality game [64].

In our study, we use the WoZ method for 2 main objectives. First, to simulate the Medical Assistance and Rehabilitation Intelligent Agent (MARIA) prototype to validate and improve our use of Alderian theory in designing the CA’s workflow for medication adherence. Second, to test and improve the overall user experiences using MARIA, which engages users in adherence to medication.

### Goal of Study

The goal of our study is to conceive a design method for developing CA for patients’ use in medication adherence, using design thinking as the main process for gathering requirements, prototyping, and testing.

We apply our design method in the ongoing development of MARIA, a rule-based CA.

The end goal of the study is to identify improvements in the functionality and dialogue construction of MARIA. This could be applied to leverage existing technologies that use CA or chatbot, such as ChatGPT or AWS Lex, to adapt it within a health care setting.

In this paper, we report on the results of our observation study protocol applying our design method for CA development.

## Methods

### Design Thinking Processes

#### Methodology Processes

**Figure 2:**
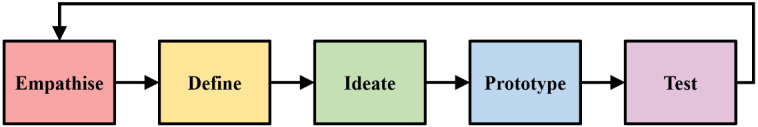
Design thinking methodology.

The design thinking methodology consists of 5 processes (phases) [65]: empathize, define, ideate, prototype, and test, as shown in Figure 2.

The process can be nonlinear and iterated until the best solution to the problem is achieved [66]. In our research, we conducted 1 iteration of the design thinking process to improve our prototype design.

#### Empathize

Constructing empathy to understand the stakeholders and their problems is essential in human-centered consideration and is the core of the design thinking process [67]. In our research, we conducted the steps outlined in Textbox 1 to gather detailed information to understand the problem and stakeholders’ needs better.

**Steps to gather information to understand the problem and stakeholders’ needs better.**

- Review of the current state of the system

We reviewed the previous achievements of Medical Assistance and Rehabilitation Intelligent Agent’s (MARIA) design to observe the relevant context, including the tasks accomplished by the Monash research team in this project [33,68].

- Work practice observations and interviews

As MARIA aims to perform as a personal nurse assistant to motivate patients about medication adherence, we studied the work procedures for managing patients with heart failure (HF) in Malaysian cardiac centers. We use ethnographic studies and interviews as a method to gain insights into the work practices in the management of patients with HF [33].

- Design thinking meeting

We organized a design thinking meeting to collect stakeholders’ requirements and practice knowledge about encouraging and educating patients with HF to adhere to their medication. We refer to the requirements method in the work by Abdullah et al [33] where several iterations of meetings take place.

The meeting involves direct and indirect stakeholders, those who will be using it directly (patients) and those who are part of the patient management team (pharmacists and specialists). Specifically for our work, we involved the supervisor from Monash Malaysia as the project lead, at least 3 medical doctors from the Malaysian cardiac centers, 2 pharmacists, 3 developers, and 1 student researcher from Monash Australia as the MARIA conversational agent designer. The meetings were conducted iteratively until all team members reached common ground on the pain points of HF management, as well as the challenges faced by health care practitioners in ensuring medication compliance in these patients. Every meeting was recorded for further analysis by the researcher and validated by the team.

#### Define

Based on the requirements of stakeholders’ needs and the research context, the “Define” stage identifies the problem and the factors contributing to this problem [67]. We applied the thematic, qualitative analysis approach to capture stakeholders’ essential requirements and the core issue [69]. We created the edited transcription to omit the unnecessary content in the recorded meeting conversations to help us retain the recording quality and capture the critical information in the collected data [70]. We marked the latent codes in our meeting transcription to demonstrate the underlying themes from the interpretative level [69]. Then, we analyzed and categorized the thematic codes to define the critical problem and stakeholders’ expectations in MARIA’s expanding design.

#### Ideate

The conceptual solution to the defined problem is generated in the ideate phase, and the brainstormed outcomes are the potential source for building the prototype [66]. We integrated the literature review of the relevant studies, the context learning of the cardiac center’s work procedures, and the thematic analysis of stakeholder’s requirements, and then visually demonstrated our design concept in the MARIA Interaction Protocol for Motivating Patients. We used a workflow diagram to display our protocol. The diagram can illustrate the step-by-step procedure for completing a task in a logical sequence, define how information and responsibility are transferred between parties during the task, clearly indicate the beginning and end of the process, and display parallel paths reflecting the consequences of different decisions or alternative options [71]. Our protocol contained the set of activities that MARIA should carry out and follow during the interaction with patients with HF. The activities were designed to ensure MARIA performs the role of personal nurse assistant to encourage and educate patients about medication adherence from home and reduce rehospitalizations and medical staff’s workload.

#### Prototype

A prototype is a quick and cost-saving conceptual model built to obtain valuable user feedback for further optimization considering the final product’s practical application [67]. It leads the design closer to the final solution [66]. Based on our proposed protocol, we prototyped the conversational templates using the decision tree method. This method is commonly adopted in designing the data-mining algorithm for predicting multiple target variables [72]. We designed our decision-tree templates to suit the future programming of the MARIA conversational system [68]. Encouragement and education strategies were included in the conversational templates to enhance patients’ confidence in medication adherence. The design also covered the reinforcement of MARIA’s human-likeness and reliability to enhance patients’ user experience and trust for the long-term use of the MARIA application.

#### Test

The test stage provides another opportunity to apply empathy by comparing the user feedback and the initial understanding of the requirements. It evaluates whether the defined problem has been successfully addressed and delivers the information for refining the prototype [66].

We use an observational study protocol to design the WoZ method and a user satisfaction scoring test at this step.

WoZ was used to simulate MARIA to validate and improve our use of dialogue designs. The user satisfaction scoring test, by contrast, was used to evaluate the engagement of patients with the MARIA prototype (Multimedia Appendix 1).

### The WoZ Method for the Observational Study Protocol

Our conceived WoZ in an observational study protocol was designed to simulate the interaction of MARIA with participants, aiming to validate (testing) and refine template responses (ie, CA’s workflow dialogue) while gathering user experience feedback.

Given that the aim of using WoZ was ultimately to improve the design of a rule-based CA, we did not control for participants’ beliefs about whether they were interacting with a real person or whether the study procedure (ie, the MARIA prototype) was successful. Instead, participants interacting with MARIA believed it was autonomous. Our researcher (CHY), acting as the wizard, operated MARIA from another room.

The number of participants varies from one work to the other with no consensus on the ideal number of participants when used in a WoZ method. For example, the work of Bonial et al [73] involved 10 participants in the study. On the other hand, Nielsen and Norman’s [74] recommendation for usability testing, which the WoZ also falls into, required 5 participants to test. By contrast, in requirements elicitation [75], there are no specific guidelines for the number of persons required; it can vary from 2 to 12 persons.

Given that there is no agreement on the number of sample sizes, we follow a qualitative study recommendation of 20 samples [76] as an initial sample size. Furthermore, because the protocol is designed as an interactive process, researchers may stop to recruit further sample size when analysis suggests that data are saturated (ie, not many differences in the responses at a certain point).

### Ethical Considerations

MARIA_PRO_VER_3_190122 is registered with the Malaysia Medical Ethics Committee. The Medical Research Ethics Committee, the Ministry of Health Malaysia, approved the study with the registration number NMRR-21-1388-60672 (IIR). Patients provided informed consent before their involvement in the study and consented to use their data for analysis. The patients were provided compensation after completing the WoZ study.

### Privacy and Confidentiality Protection

Participant names for this research have been deidentified and linked only with a study identification number. Therefore, the research did not identify the participant’s identity and instead used anonymized identification numbers on all the data sets. All data are stored in Monash University Malaysia REDCap secured cloud and kept for 3 years. Participants can write to the investigators to request access to study findings.

### Study Procedure

**Figure 3:**
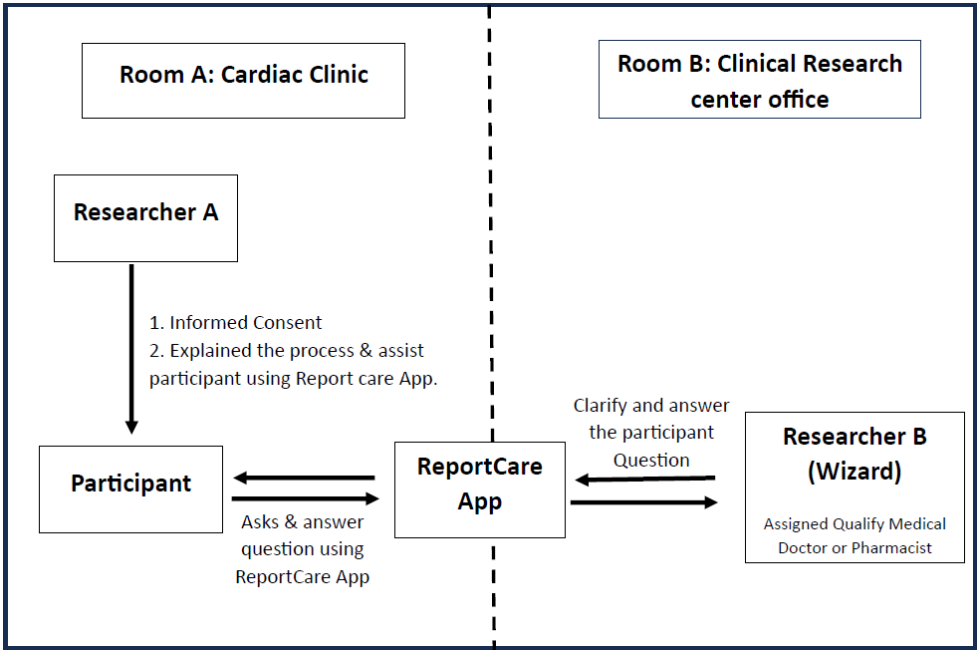
Overall Wizard of Oz study procedure.

During the recruitment and study period, there were 2 researchers, researcher A and researcher B, each located in separate facilities. Researcher A was based in the cardiac clinic, whereas researcher B operated from the Clinical Research Center (CRC) office. Participants were assigned to the cardiac clinic with researcher A. Researcher B worked from the CRC office (refer to Figure 3).

The study protocol allowed only 1 participant at a time in each room, with each session being conducted sequentially, 1 participant following another. Textbox 2 provides an explanation of the roles and responsibilities of the researcher and participant. Part A details the roles of researcher A and the participant, while part B outlines the responsibilities of researcher B.

**Roles and responsibilities of the researcher and participant.**

- Part A: role of researcher A and participant/setting: room A—cardiac clinic

- Informed consent process:
  Researcher A explained the details of the research and the participant signed the consent.
  The participant is provided with a unique ID for deidentification purposes.
- Explanation of the process and assisting participants in using the web app:
  The participant will be seated in a room and given a smartphone with the web app preinstalled.
  Researcher A will explain how to use the web app and Medical Assistance and Rehabilitation Intelligent Agent (MARIA), the messaging chatbot as a self-management tool, in a home setting.
  The participant will log into the web app using the unique ID provided.
- Given scenarios:
  Researcher A gives a set of written scenarios to participants (for participants to recall their usual symptoms or signs that they experienced) and the common questions or clarification participants would like to ask MARIA related to the given scenario.
  The participants will respond with their questions based on the scenario using the web app messaging feature.

- Part B: role of researcher B (to role-play the wizard) delegated to a qualified medical doctor and pharmacist/setting: room B—Clinical Research Center office

- Researcher B will be provided with the participant ID and basic information (sociodemographic and medication history).
- Researcher B will refer to the Heart Failure Clinical Practice Guidelines [23] and the Pharmacy Practice and Development Division, the Ministry of Health Malaysia [77], and the Protocol for the Medication Therapy Adherence Clinic [24]. In particular, the researcher will follow:
  The workflow on therapy medication protocol adherence for furosemide titration, including management of side effects.
  The workflow for general inquiries on the medication side effects of furosemide and beta-blockers [78].
  The workflow on the management of symptoms and signs.
- According to the standard workflow, researcher B will respond to participants via the messaging chatbot provided in the ReportCare app.
  Pharmacists and medical doctors will respond to drug- or clinical-related questions such as medication titration, drug dosage, frequency, side effects, and drug interaction.

### Recruitment

Study participants were recruited from the Hospital Queen Elizabeth II, Sabah in Malaysia. The participant recruitment process was from June 2022 to November 2022.

The recruitment process followed the Malaysian Good Clinical Practice guidelines. The participants for this study were identified by CHY (principal investigator) at the HF clinic. During the consultation, the investigator explained the study to the patients and provided the consent form. If the patient fulfilled the inclusion and exclusion criteria, they were given sufficient time to read, discuss the study, and ask any questions. All questions were answered by the investigator. After addressing the patient’s concerns, the patient signed the consent form.

### Study Population

The study population included patients with chronic HF who were currently being followed up at the Cardiology Department Outpatient Clinic in Hospital Queen Elizabeth II. The inclusion criteria were: (1) age above 18 years, (2) diagnosis of chronic HF for at least one year, (3) history of symptomatic HF, (4) ability to write and speak Malay and English, (5) ability to type and use mobile app messaging, and (6) ability to comply with the protocol.

The exclusion criteria were as follows: (1) the presence of a clinical condition that would interfere with participation in the interview and (2) mental or legal incapacitation preventing the patient from providing informed consent.

### Sample Size

Typically, the sample size is small at the beginning, as the goal is to explore the system. With each improvement, the process continues until an acceptable usability score or set of requirements is achieved [73-75].

As stated in the “The WoZ Method for Observational Study Protocol” section, given the lack of agreement on sample size, we follow a qualitative study recommendation of 20 samples [76].

We use usability scoring as a quantitative standard to determine the acceptability of the system’s design before proceeding with implementation. Hence, for the initial sample size, we used a convenience sampling method, recruiting a minimum of 20 patients for the study.

- Ten participants can speak and write the Malay language.
- Ten participants can speak and write the English language.

### Study Duration

The total time required for each participant to participate in the study was a maximum of 1 hour.

### Wizard Protocol

#### Overview

Below, we share an excerpt from MARIA’s workflow protocol for goal setting, daily monitoring, and goal completion.

#### Wizard Preparation

**Figure 4:**
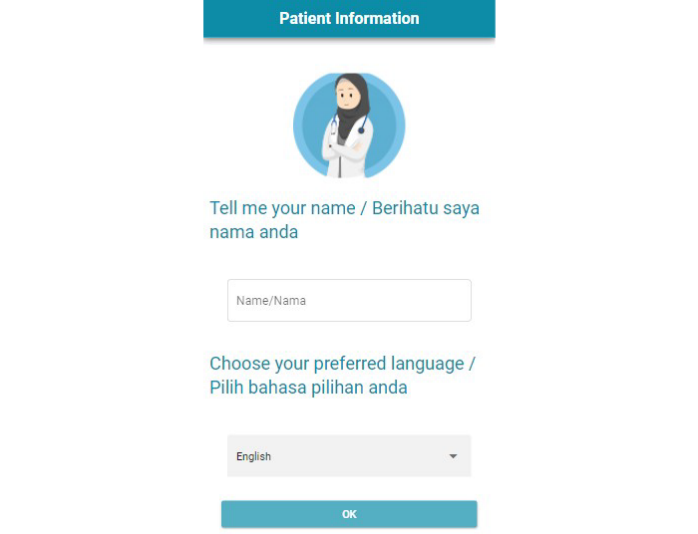
Screenshot of the app displaying the log-in interface including the language selection feature.

The wizard (researcher B) launched the web app (Figure 4) before the patient, entered “MARIA” as the name, and selected either English or Malay based on the patient’s preferred language. The participant then waited to launch the web app (refer to participant protocol). The wizard entered the participant’s name, after which the web app redirected to the chatbox, where the participant entered their name(s).

#### Conversation Protocol

**Figure 5:**
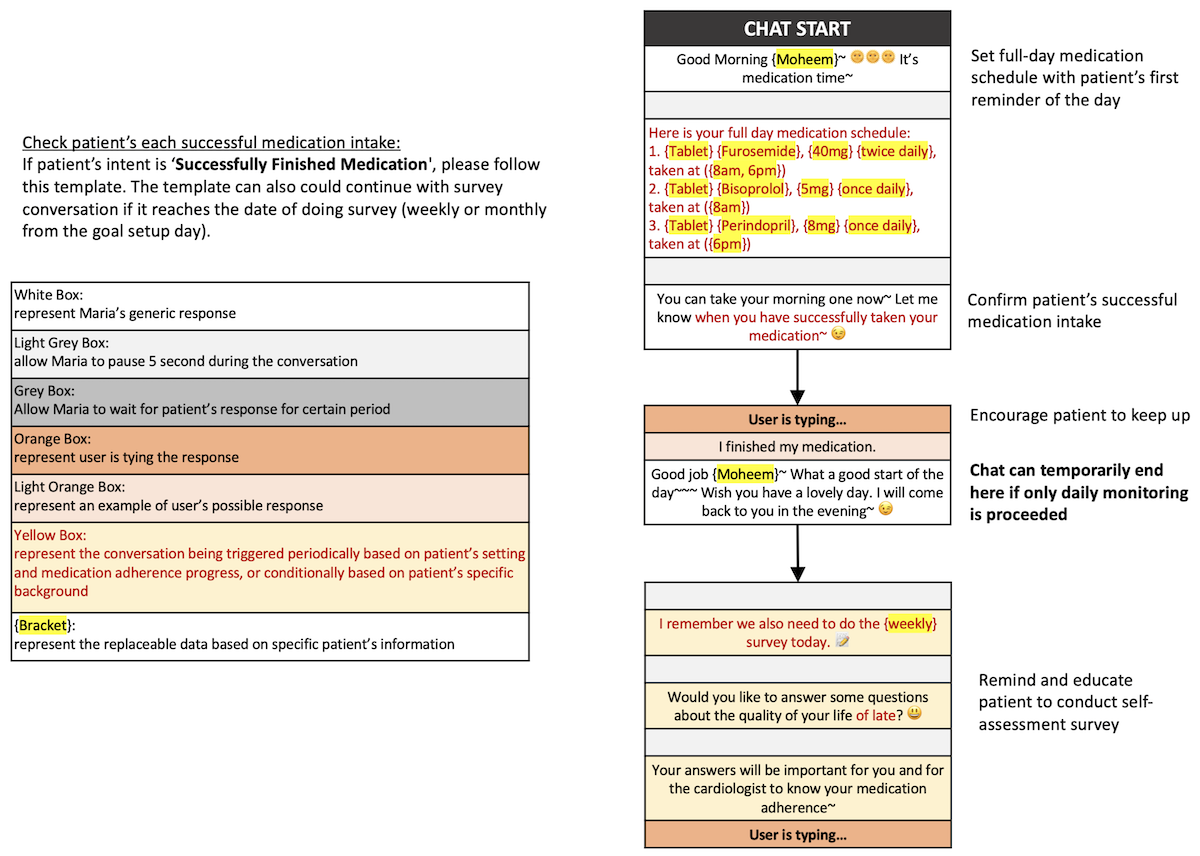
Overview of the conversation protocol as followed by the Wizard throughout the study.

In this study, the participant will ask questions based on the conversation flowchart (Figure 5). If the question follows the predefined flow, researcher B (wizard) will respond or ask a follow-up question accordingly. However, if the question or response deviates from the flow, researcher B (wizard) will intervene, providing an appropriate response or asking a relevant question to steer the conversation back on track. This intervention ensures that the discussion remains focused and addresses any inquiries outside the predefined flow. Researcher B (wizard) will continue following the conversation flowchart and await the participant’s responses.

#### Participant Protocol

Researcher A is responsible for obtaining participants’ consent and collecting their basic demographic and medical history information, which is then provided to the wizard (researcher B) for further analysis.

Researcher A also assists participants in launching the web app on their mobile devices. Once participants enter the chat room, they can ask questions or respond using the web app interface.

Before participants begin their conversation with the wizard, researcher A explains the research process, which is divided into 3 parts: part 1 (goal setting), part 2 (daily monitoring), and part 3 (goal completion). Each part is explained in detail to the participant.

In part 1 (goal setting), researcher A highlights the importance of goal setting, while the wizard (researcher B) follows the predefined flowchart to assist participants in setting up medication reminders and emergency contacts.

In part 2, researcher A presents scenarios related to medication adherence, such as remembering or forgetting to take medication. Participants respond to these scenarios, and the wizard (researcher B) provides appropriate replies based on their answers.

In part 3, the wizard (researcher B) follows the conversation flowchart to ask participants about their quality of life and updates the relevant information accordingly.

### Conversation Analysis

We developed a coding guideline for analyzing the utterances, as detailed in Textbox 3.

The researcher tested the coding guideline before providing it to the clinical researcher, who then used it to analyze the collected data from the study participants.

**Coding guideline.**

Objectives of coding

- To identify speech act verbs of each utterance
- To identify turn-taking
- To identify which workflow was used to map each utterance
- To annotate the workflow part that has been modified

Instructions

- Follow the sample provided for annotating each individual’s chat logs.

Workflow

Each utterance is mapped to the workflow that was used by the wizard as follows:

- If it is not in the workflow, simply annotate with N/A (not applicable)
- If it is part of the workflow, simply annotate the corresponding workflow reference (eg, “Workflow: Daily Monitoring”)
- If it is part of the workflow but was modified during the study, add the remark “Modified” in the remark column.

Speech act definition and example of annotation

A speech act is an utterance that serves a communicative function. We perform speech acts when we offer an apology, greeting, request, complaint, invitation, compliment, or refusal. A speech act may consist of a single word, such as “Sorry!” to express an apology, or multiple sentences, such as “I’m sorry I forgot your birthday. It just slipped my mind.” Speech acts occur in real-life interactions and require not only linguistic knowledge but also an understanding of appropriate language use within a given cultural context.

Here are some examples of speech acts we use or hear every day:

*Greeting:* “Hi, Eric. How are things going?”

*Request:* “Could you pass me the mashed potatoes, please?”

*Complaint:* “I’ve already been waiting three weeks for the computer, and I was told it would be delivered within a week.”

For the speech act definition, we refer to the work of Vanderveken [79].

Topic

The topic, in essence, is what is being communicated in a sentence. You may use the topics identified by the template. If none of the provided topics fit the chat you are analyzing, you may define a new topic.

Turn-taking definition and analysis

- Turn-taking occurs in a conversation when one person listens while the other speaks. As the conversation progresses, the roles of listener and speaker are exchanged back and forth in a cyclical manner.
- Analyzing turn-taking is essential to assess whether both participants are engaged in communication. It can be examined using different units of measurement, such as adjacency pairs, continuing turns, and intervention turns.
- For our dialogue modeling, we use adjacency pair turn-taking as the unit of analysis. Adjacency pairs consist of 2 utterances produced by different speakers. To form an adjacency pair, there must be at least two speakers. In adjacency pairs, the first utterance—known as the first pair part—requires a response, while the second utterance—known as the second pair part—serves as the response to the first.

Here are some examples:

**Question and answer**

*Speaker 1:* “Where’s the milk I bought this morning?”

*Speaker 2:* “On the counter invitation.”

**Invitation and Acceptance**

*Speaker 1:* “I’m having some people to dinner on Saturday, and I’d really like you to come.”

*Speaker 2:* “Sure!”

### User Satisfaction Scoring Test

**Figure 6:**
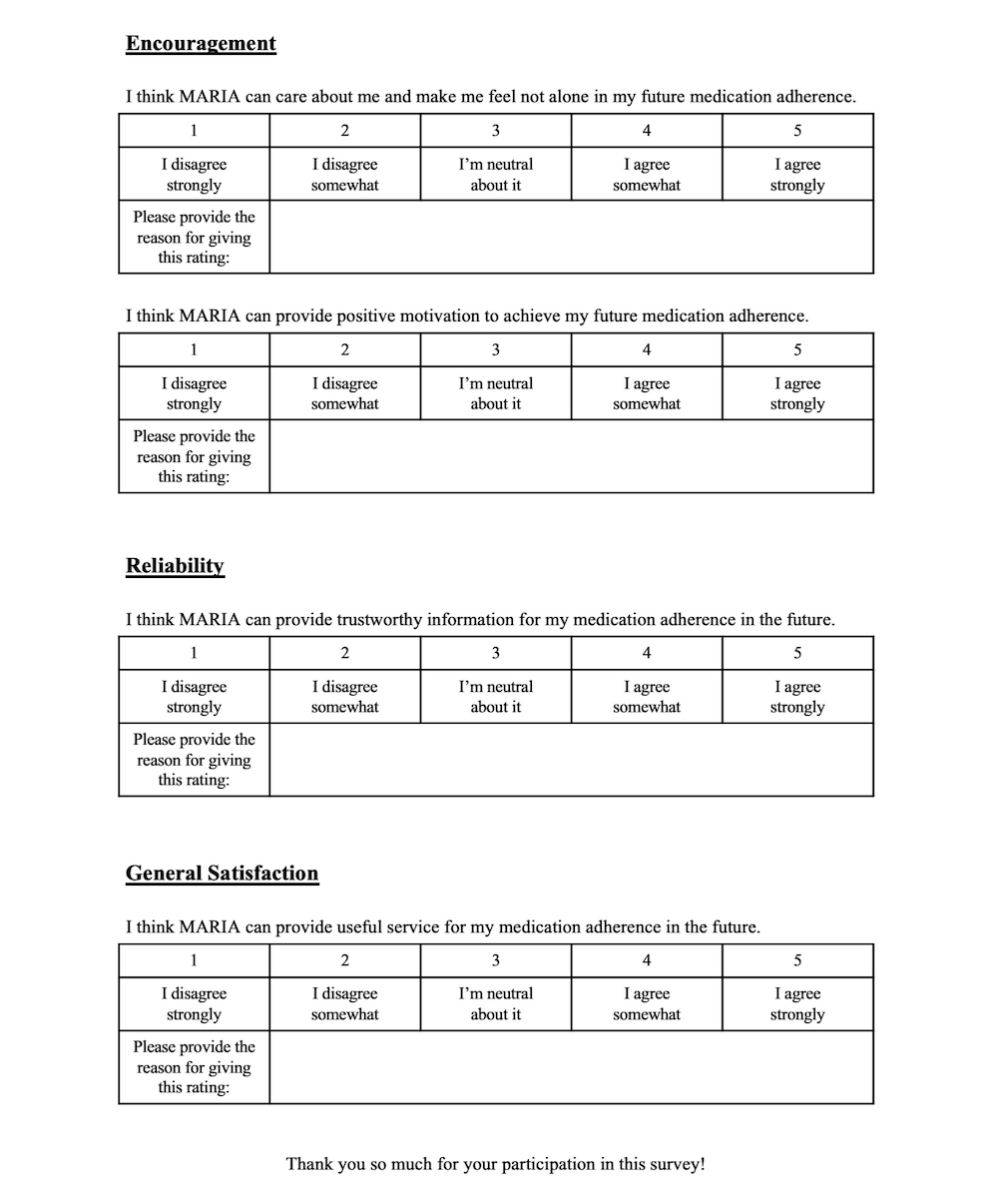
An excerpt from the usability evaluation survey.

We used Hoffman et al’s [80] evaluation of user trust in AI systems. Our questionnaire includes Likert-scale questions rated from 1 to 5, where 1 represents “I disagree strongly” and 5 represents “I agree strongly.” Additionally, we included open-ended questions to understand the reasons behind the given ratings. The questionnaire focuses on evaluating our conversational template design from various aspects (Figure 6), including human-likeness.

For human-likeness, which encompasses MARIA’s natural human language use, personality consistency, and expressed emotions, we define the criteria used for usability scoring.

- Educational strategies: Evaluate MARIA’s effectiveness in tutoring patients on completing daily medication intake and providing appropriate knowledge to clarify medication use and side effects.
- Encouraging strategies: Assess MARIA’s ability to offer care, support, and positive reinforcement to motivate patients toward medication adherence.
- Reliability: Reflects patients’ trust in the accuracy of the information provided by MARIA during interactions.
- General satisfaction: Captures the overall impression of MARIA’s conversations and their applicability.

## Results

### Evaluation of MARIA’s Conversational Design and Its Implications for Medication Adherence

The evaluation outcomes indicate that our conversational template design generally met the needs of stakeholders, including end users, patients, and pharmacists. MARIA’s natural language interactions, along with its encouragement and education strategies, are expected to support medication adherence among patients with HF in the future. However, the study also highlighted concerns regarding system liability and raised discussions on the extent to which MARIA should provide educational content on medication interactions and side effects in response to patient inquiries.

### Evaluation

#### Coding Analysis

Each logged utterance was transferred into an Excel sheet (Microsoft Corporation). Independent coders (ie, clinical researchers) conducted the coding analysis based on the provided instructions (Multimedia Appendix 2). An example of the coding analysis is presented in Multimedia Appendix 3.

On average, study participants engaged in 30 interactions with the wizard, with a turn-taking ratio of 4:1 between the wizard and participants per topic. This pattern indicates that participants primarily engaged in question-answer exchanges with the wizard. The topics and speech acts used in the dialogue aligned with psychoeducational therapy theory, as evidenced by annotations of speech acts such as suggestions, support, and applause. However, having the wizard simulate MARIA revealed gaps in the workflow, including challenges in addressing negative responses, the appropriate use of emoticons, and the system’s feedback mechanism during turn-taking delays.

Regarding topics, patients were most interested in asking about medication interactions and side effects. However, given MARIA’s high average turn-taking per study participant, patients provided feedback suggesting that chat messages should be more concise—ideally limited to a single sentence. Longer messages often cause patients to lose track of the topic, requiring them to re-read the content for clarity.

#### Usability Scoring

Table 1 presents the evaluation results for the usability scoring of the MARIA CA design, including demographic data of the study participants.

The human-likeness of interactions with MARIA received a median score of 4.75 out of 5. However, MARIA’s personality scored lower, with a median of 3.8. In terms of natural language use, patients generally felt that conversing with MARIA resembled real human communication (question 1). One participant noted, “I am aware that I’m chatting with an AI. However, most responses were similar to what I would expect from a human.”

However, MARIA’s demonstration of personality and emotions (question 2) received the lowest rating in the evaluation. While the designed conversations made patients feel friendly and cared for, one patient noted a lack of distinct character in MARIA as a health assistant.

Regarding guiding patients to follow the medication routine (question 3), all fictional patients believed that MARIA’s tutoring strategy would effectively support future medication adherence.

Feedback indicated that the educational content provided by MARIA was clear and easy to understand, with its knowledge-sharing approach helping patients learn about medication functions (question 4).

Additionally, in terms of encouragement strategies, fictional patients confirmed that MARIA’s conversations were highly encouraging, fostering a sense of support and assisting with medication adherence (question 5).

“It is a good feeling if you open your phone, and someone (AI) keeps reminding you about your medication,” one patient commented, highlighting MARIA’s role in fostering adherence. Additional feedback reinforced MARIA’s supportive nature, with remarks such as “MARIA is supportive of me, and I feel motivated every day” and “MARIA is very perseverant” (question 6).

Regarding reliability (question 7), 1 patient expressed trust in MARIA for medication management, while another noted the need to confirm information with a doctor. Despite this, MARIA received an average satisfaction score of 4.5 (question 8), with patients affirming its effectiveness in reminding them to take their medication on time.

From a patient safety perspective, the wizard, played by the pharmacist, played a crucial role in defining the extent to which a CA could communicate on behalf of a health care provider regarding medication adherence. Initially, the study included a workflow for educating patients about medication side effects. However, concerns arose about the implications of automating responses by retrieving drug side effect information from web-based sources. Based on these concerns, the decision was made to remove the workflow for medication side effects to ensure accuracy and patient safety.

**Table 1:** Participants’ demographic data and usability evaluation results.

Demographic				Values
**Sex, n**				
	Male			15
	Female			5
Age (years), mean				49
**Human-likeness**				
	**I think MARIA^a^ can talk like a real person, n**			
		I disagree strongly	0	
		I disagree somewhat	0	
		I am neutral about it	0	
		I agree somewhat	5	
		I agree strongly	15	
	**I think MARIA can show her personality and emotion during the conversation, n**			
		I disagree strongly	2	
		I disagree somewhat	2	
		I am neutral about it	2	
		I agree somewhat	6	
		I agree strongly	8	
**Education**				
	**I think MARIA can guide me to complete my daily medications in the future, n**			
		I disagree strongly	0	
		I disagree somewhat	0	
		I am neutral about it	1	
		I agree somewhat	4	
		I agree strongly	15	
	**I think MARIA can remove my misunderstanding about medication use and side effects, n**			
		I disagree strongly	0	
		I disagree somewhat	0	
		I am neutral about it	4	
		I agree somewhat	7	
		I agree strongly	9	
**Encouragement**				
	**I think MARIA can care about me and make me feel not alone in my future medication adherence, n**			
		I disagree strongly	0	
		I disagree somewhat	0	
		I am neutral about it	2	
		I agree somewhat	8	
		I agree strongly	10	
	**I think MARIA can provide positive motivation to achieve my future medication adherence, n**			
		I disagree strongly	0	
		I disagree somewhat	0	
		I am neutral about it	2	
		I agree somewhat	8	
		I agree strongly	10	
**Reliability**				
	**I think MARIA can provide trustworthy information for my medication adherence in the future, n**			
		I disagree strongly	0	
		I disagree somewhat	0	
		I am neutral about it	3	
		I agree somewhat	8	
		I agree strongly	9	
**General satisfaction**				
	**I think MARIA can provide useful service for my medication adherence in the future, n**			
		I disagree strongly	0	
		I disagree somewhat	0	
		I am neutral about it	1	
		I agree somewhat	6	
		I agree strongly	13	
**Background history**				
	**Disease, n**			
		Ischemic dilated cardiomyopathy	10	
		Nonischemic dilated cardiomyopathy	10	
	**New York Heart Association, n**			
		I	16	
		II	4	
	**Education level, n**			
		Primary	1	
		Secondary	9	
		Higher level education/tertiary	8	
		Post degree	2	
	**Occupation, n**			
		Unemployed or pensioner	4	
		Self-employed	4	
		Housewife	3	
		Engineer	2	
		Administrative	4	
		Teacher	2	
		Designer	1	
	**Ethnicity, n**			
		Malay	2	
		Chinese	2	
		Bumiputra Sabah	16	

^a^MARIA: Medical Assistance and Rehabilitation Intelligent Agent.

## Discussion

### Principal Findings

The design thinking method provided an iterative process that actively engaged end users from the early stages of developing the MARIA prototype, a rule-based CA.

The involvement of a multidisciplinary group of stakeholders during the ideation phase facilitated the early conceptualization of the dialogue workflow, guided by psychoeducational theory—specifically, Adlerian therapy.

During the testing phase, the WoZ methodology and user satisfaction scoring were integrated into an observational study protocol. This approach enabled the collection of simulated real-world dialogues between patients and the MARIA prototype, operated by the wizard (pharmacists), allowing for iterative refinement and validation of the CA’s conversational design.

The dialogues generated between the wizard (pharmacists) and the patients were systematically analyzed using coding analysis. This approach enabled the categorization of utterances into dialogue workflow components, speech acts, and topics, facilitating a structured evaluation of MARIA’s conversational framework.

Speech acts—such as informing and expressing gratitude—were examined in relation to their associated topics and mapped to the dialogue workflow. This mapping validated the practical application of Adlerian theory, demonstrating its effectiveness in guiding the wizard to motivate patients toward medication adherence. Furthermore, the user satisfaction scores from patients confirmed the feasibility of applying Adlerian theory within the medication adherence dialogue workflow.

Additionally, the analysis identified instances where patient-initiated utterances—either new topics or responses—were not covered in the predefined dialogue workflow. These gaps highlighted areas for further refinement in MARIA’s conversational design.

Building on this, the coding analysis reinforced the critical role of the wizard—played by an appropriate expert, in this case, pharmacists—as a key stakeholder in shaping how MARIA’s dialogues should be modeled. For instance, it became evident that advising on medication interactions and side effects cannot be delegated to the CA, as these responses require human expertise to ensure patient safety. This insight guided the identification of various scenarios that must be accounted for from a patient safety perspective when designing MARIA’s dialogue framework.

Furthermore, the user satisfaction scoring on human-likeness and trust highlighted the necessity of ensuring that MARIA’s dialogues and use of emojis align with professional communication standards. Patients expressed a greater willingness to trust MARIA’s advice on medication adherence when interactions were conducted professionally. This finding underscores the importance of designing CA interactions that balance humanlike engagement with a level of professionalism that fosters trust and credibility.

### Improvements

Through further analysis of the WoZ chatting history, we identified specific areas in MARIA’s designed conversations that required optimization. These insights guided refinements to the current template design, ensuring a more effective and user-centered interaction experience. Based on these findings, we iterated on the conversational templates and provided the final version to the MARIA research team for future implementation.

In specific interactions, the MARIA medical team, drawing from their practical experience with patients with HF across various age groups, suggested that formal language use may be more suitable than casual language.

The use of words such as “cool” in MARIA’s responses may create a more relaxed conversational style, which could be effective for younger patients but may not align with the preferences of older patients. Replacing “cool” with “excellent” could be more universally accepted across all age groups.

Specific messages should be designed to emphasize patients’ responsibility in self-managing medication adherence. For example, MARIA should educate patients that they are not merely completing a task instructed by MARIA but actively working toward their own health goals. The messaging should reinforce that patients are empowered to take charge of their health, while MARIA serves as an assistant, supporting them in improving their health status.

Educating patients about medication in advance can help alleviate their concerns. MARIA should provide reference links to information on medication and HF for patients to review before following their medication plan. This approach can enhance patients’ understanding of proper medication use, improve their awareness of potential side effects, and reduce the risk of misunderstandings about treatment effectiveness. Additionally, it may help prevent severe emergencies.

### Outcomes

The evaluation outcomes indicate that our conversational template design generally met stakeholders’ needs. MARIA’s natural language conversations, along with its encouragement and education strategies, are expected to support patients with HF in adhering to their medication. We identified several modifications that could enhance the applicability of the current conversational templates.

### Limitations

This section discusses the study’s limitations and directions for future research. In this study, we were constrained by the absence of a database containing basic medication knowledge and patient stories of successful adherence to HF medication at the prototype stage. Future development should focus on enriching MARIA’s knowledge database to better support the designed education and encouragement strategies. The database should include comprehensive medication information from reliable sources and feature shared experiences of patients with HF who have successfully adhered to their treatment. Additionally, MARIA should be trained to provide tailored encouragement for patients facing various challenges in medication adherence. While linking to existing reputable HF associations worldwide is essential, collecting and curating real-life encouragement stories at the local level could improve cultural relevance and applicability. Furthermore, the study’s participant pool was predominantly male, with limited female representation. This gender imbalance may affect the generalizability of the findings, and future research should ensure a more balanced representation to strengthen the applicability of the results.

Furthermore, as this is the initial stage of development, our focus was on covering a broad range of aspects rather than deeply exploring anthropomorphism. In future development stages, we plan to conduct a more detailed evaluation of anthropomorphism to enhance MARIA’s human-like interactions.

### Conclusions

This study demonstrated that applying design thinking processes provides practical, interactive steps to engage users early in the design, prototyping, and testing of a CA for supporting patients in self-managing their medication. Furthermore, using the WoZ simulation method within an observational study protocol at the testing stage proved to be a valuable approach for refining the CA’s interaction model, validating its functionality, and assessing system reliability, user expectations, and potential constraints. Results from the WoZ simulation and user satisfaction scores indicated that MARIA is a feasible and acceptable medication assistant CA. Additionally, patients expressed a general willingness to integrate MARIA into their daily routines to enhance medication adherence at home.

## Appendix

Multimedia Appendix 1 User satisfaction of MARIA Interaction feedback. MARIA: Medical Assistance and Rehabilitation Intelligent Agent.

Multimedia Appendix 2 Samples of Wizard of Oz utterance analysis.

Multimedia Appendix 3 Excerpt from the coding analysis.

## Abbreviations

AI: artificial intelligence
CA: conversational agent
CRC: Clinical Research Center
DH: digital health
HF: heart failure
MARIA: Medical Assistance and Rehabilitation Intelligent Agent
WoZ: Wizard of Oz

## Data Availability

The manuscript provides the chat data. The MARIA prototype and the code are available for other researchers to use upon request. Researchers may also contact the main author to learn more about the design process.
