# Supplementary material for: MARIA (Medical Assistance and Rehabilitation Intelligent Agent) for Medication Adherence in Patients With Heart Failure: Empirical Results From a Wizard of Oz Systematic Conversational Agent Design Clinical Protocol
Source: JMIR Cardio. 2025 Apr 10;9:e55846. doi: 10.2196/55846 (PMC12022519; doi:10.2196/55846)
Supplement: Multimedia Appendix 1 [file cardio_v9i1e55846_app1.pdf]

APPENDIX 1 - User Satisfaction of Maria Interaction feedback

Participants	Date	Gender	Age	Human-likeness			Education			Encourgement			Reliability		General Satisfaction		Others comment:
				a) I think Maria can talk like a real person	b) I think Maria can show her personality and emotion during the conversation	Reason/comments	a) I think Maria can guide me complete my daily medication in the future	b) I think Maria can remove my misundersatnding about medication use and side effect.	Reason/comments	a) I think Maria can care about me and make me feel not alone in my future medication adherence	b) I think Maria can provide positive motivation to achieve my future medication adherence	Reason/comments	a) I think Maria can provide trustworthy information for my medication adherence in the future	Reason/comments	a) I think Maria can provide useful service for my medication adherence in the future	Reason/comments	
1	29-Jun-22	Female	44	5	5	1. put emojis<br>2. shorten the sentences	5	5		5	5		5		5		
2	30-Jun-22	Male	61	4	1	1. questions are good. Respond is slow. Answer options are not listed. 2. No emotional statement	5	3	Yes	5	5	good reminder system	5		5		
3	1-Jul-22	Female	56	5	4	1. natural wording<br>2. Need improvement by doing icon	5	4		4	4		4		4		1. should design or develop a voice/sound notification for medication reminder
4	14-Jul-22	Female	37	5	4		5	4		4	3	1. Not all patients truthful with their medication adherance, this MARIA will not detect if the patient is	4		3		
5	22-Jul-22	Male	38	5	5		5	5		5	5		4		5		1. Need 'typing..' status 2. Malay language can be understood 3. Useful for reminding to take medication 4. Questions easy to understand
6	25-Jul-22	Male	72	5	5		5	5	1. With 2-way traffic	5	5	1. by me asking a lot of questions too	5		5		1. Easy to understand
7	26-Jul-22	Female	37	5	5		5	4		4	4		5		5		1. How Maria remind? Through notifications? 2. Voice function might be useful for elderly
8	15-Aug-22	Male	37	4	3		5	5		5	5		5		5		1. Option for camera to send document 2. Emergency button for nearby hospital
9	22-Sep-22	Male	67	5	5		5	5		5	5		5		5		
10	27-Sep-22	Male	53	4	2		3	3		3	4		4		4		
11	30-Sep-22	Male	52	5	5		4	5		4	4		3		5		
12	7-okt-22	Male	36	5	4		5	4		4	5		4		5		1. Malay language easy to understand 2. Cannot reply to specific message (like whatsapp)
13	19-Okt-22	Male	49	5	2	1. the conversation are professional	5	3		3	5		3		4		
14	19-Okt-22	Male	49	4	4		4	4		4	4		4		4		
15	21-Okt-22	Male	39	5	1	1. feel like a friend that can remind to take medicine	5	5		5	5		5		5		1. Malay language easy to understand
16	3-Nov-22	Male	54	5	4	1. show emotion during forgot to take medicine	5	5		5	5		5		5		1. Malay language easy to understand
17	4-Nov-22	Female	42	5	5	1. Emojis shows like a real person 2. Can understand what patient trying to say	5	3	1. Notifications with sound in future	5	5	1. Positive motivation to take medicine	5		5	1. Useful for busy people and in rural areas	
18	11-Nov-22	Male	59	5	4		4	4		4	3		3		4		
19	16-Nov-22	Male	60	4	3		4	4		4	4		4		4		
20	17-Nov-22	Male	38	5	5		5	5		5	5		4		5		

Scale	Represents statement
1	I disagree strongly
2	I disagree somewhat
3	I'm neutral about it
4	I agree somewhat
5	I agree strongly
