# Supplementary material for: MARIA (Medical Assistance and Rehabilitation Intelligent Agent) for Medication Adherence in Patients With Heart Failure: Empirical Results From a Wizard of Oz Systematic Conversational Agent Design Clinical Protocol
Source: JMIR Cardio. 2025 Apr 10;9:e55846. doi: 10.2196/55846 (PMC12022519; doi:10.2196/55846)
Supplement: Multimedia Appendix 2 [file cardio_v9i1e55846_app2.pdf]

APPENDIX 2: Samples of Wizard of Oz utterance analysis

Table 1: Utterance analysis for the participant A – Original Utterance is Malay language with the details of the workflow type, and speech act topic.

Name	Utterance analysis	Workflow type	Speech act-topic	Topic	Adjacency pair, use number to count how many turns M or Z makes for each topic	Notes/remarks
MARIA	Hi {Participant_A}! \n You can type in your message or record your Utterance voice.\n (If you want to see some examples of the questions you can ask me, write 'help')",	N/A<br>N/A	describe	information on use	M-1	
{Participant_A}	Hai	N/A	answer	information on use	Z-1	
{Participant_A}	Apa ubat yang perlu saya ambil hari ini?	N/A	ask	Medication intake	Z-1	
MARIA	Berikut Hialeah pelan ubat yang telah dibuat Oleh pakar kardiologi anda untuk anda	N/A	answer	medication intake	M-1	modified
MARIA	Log in Patient name : {Participant_A}	N/A		medication intake	M-2	
MARIA	Nama ubat: Bisoprolol 5mg, satu kali sehari"	N/A		medication intake	M-3	
MARIA	Adakah anda ingin menetapkan matlamat untuk pematuhan ubat?	Goal setting	ask	Set-up goal	M-1	
{Participant_A}	Ya perlu		answer	Set-up goal	Z-1	
MARIA	Dengan menetapkan matlamat, boleh membantu anda menjalani rutin pengambilan ubat anda dengan lebih berkesan. Anda mempunyai sebulan lagi sebelum temujanji anda yang seterusnya! ðŸ™ (Slightly Smiling Face, 🙂)	Goal setting	explain	medication adherence	M-1	modified. continued from inquiry. typing delay
MARIA	Saya sangat teruja mendengarnya, {Participant_A}! Mari mulakan pelan kita ke arah kualiti hidup yang lebih baik!	Goal setting	express gratitude	medication adherence	M-1	in reply to line 13
{Participant_A}	Ok terima kasih MARIA		accept	medication adherence	Z-1	
MARIA	Pelan pengambilan Ubat untuk for {Participant_A} Tablet Bisoprolol 5mg satu kali sehari (8 pagi) Tempoh: 1bulan (29/06/2022- 29/07/2022)	Goal setting	inform	medication plan	M-1	
MARIA	Ini ialah pelan ubat yang telah ditetapkan oleh pakar kardiologi anda untuk anda. Saya pasti bahawa anda telah diberikan ubat-ubatan yang anda perlukan	Goal setting	remind	medication plan	M-2	
MARIA	Saya di sini untuk membantu dalam memastikan anda tidak terlepas pengambilan ubat anda ðŸ™ (Slightly Smiling Face, 🙂)	Goal setting	tell	medication plan	M-3	
MARIA	Ini adalah pautan untuk artikel dari laman web Persatuan Jantung Amerika mengenai maklumat ubat yang anda akan ambil. https//www.heart.org/en/health-topics/heart-attack/treatment-of-a-heartattack/cardiac-medications	Goal setting	inform	medication information	M-4	

MARIA	Berdasarkan pelan ubat ini, saya akan menetapkan peringatan untuk mengingatkan pengambilan ubat anda pada 8 pagi	goal setting	propose	medication setting	M-1	
{Participant_A}	Ok		answer	medication setting	Z-1	
MARIA	Adakah tetapan saya sesuai untuk pilihan anda? Atau adakah anda mahu mengubahnya dengan saya sekarang? ðŸ˜ (Neutral Face, 😐)	goal setting	ask	medication setting	M-1	
MARIA	Anda juga bebas untuk mengubah masa peringatan atau meningkatkan kekerapan untuk peringatan sebelum {8 pagi}	goal setting	inform	medication setting	M-2	
{Participant_A}	Ok		answer	medication setting	Z-1	
MARIA	Berdasarkan pelan ubat, saya akan menetapkan peringatan untuk mengingatkan pengambilan ubat anda pada 8 pagi.	N/A	announce	medication timing	M-1	
MARIA	Mematuhi ubat anda adalah penting. Ini akan membantu anda memperbaiki keadaan kesihatan dan kualiti hidup anda. Sebagai pembantu peribadi anda, saya akan membantu anda sepanjang masa. ðŸ™(Slightly Smiling Face, 🙂),	goal setting	explain	medication adherence	M-1	
{Participant_A}	Ok ðŸ˜Š (ðŸ˜Š Smiling Face With Smiling Eyes)		answer	medication timing	Z-1	
MARIA	Keluarga dan rakan-rakan ðŸ‘¬ ðŸ » ([illegible])juga boleh memberikan sokongan dan keyakinan kepada anda semasa pengembaraan pematuhan ubat anda	goal setting	inform	community support	M-1	not in order
MARIA	Adakah anda ingin mengaktifkan kenalan kecemasan? Ini sepenuhnya bergantung kepada anda, saya akan mengikuti keputusan anda	goal setting	suggest	contact emergency	M-1	not in order
{Participant_A}	Okay		answer	contact emergency	Z-1	
MARIA	Siapakah yang anda ingin tetapkan sebagai kontak kecemasan? Sila berikan Nama, dan Number telefon kontak yang anda ingin tetapkan.	goal setting	ask	contact emergency	M-1	modified
MARIA	Siapakah yang anda ingin tetapkan sebagai kontak kecemasan ? Sila berikan Nama, dan Number telefon kontak yang anda ingin tetapkan			contact emergency	M-2	repeated
{Participant_A}	{Emergency Contact Name},{Emergency Contact Number}		answer	contact emergency	z-1	
MARIA	Bagus! Saya akan menetapkan untuk anda sekarang. Sila sahkan dengan 'Ya'	goal setting	promise, ask	contact emergency	M-1	modified
{Participant_A}	Ya		answer	contact emergency	z-1	
MARIA	Ini adalah kenalan kecemasan anda: 1. Nama: {Emergency Contact Name} 2. Nombor untuk dihubungi: {Emergency Contact Number} Sila balas 'Ya' untuk mengesahkan atau 'Tidak' untuk mengubah	goal setting	report	contact emergency	M-1	modified
{Participant_A}	Ya		answer	contact emergency	Z-1	
MARIA	Terima kasih. Sila maklumkan kepada kontak kecemasan anda mengenai ketetapan ini	goal setting	recommend	contact emergency	M-1	modified
{Participant_A}	Ok		answer	contact emergency	Z-1	

MARIA	Adakah anda ingin melakukan tinjauan kaji selidik semasa program kita berjalan? Dalam tinjauan ini, saya akan mengemukakan beberapa soalan mudah dan umum untuk merakam maklum balas anda mengenai kepatuhan ubat. Hasil tinjauan ini dapat membantu anda untuk menilai sendiri dan memberikan maklumat kepada pakar kardiologi untuk temu janji anda yang seterusnya. Tinjauan ini dapat ditetapkan untuk setiap minggu atau setiap bulan, berdasarkan ketetapan anda. Adakah anda ingin menetapkan tinjauan secara mingguan atau bulanan?	goal setting	recommend	QAL	M-1	
{Participant_A}	Setiap mingguan		answer	QAL	Z-1	
MARIA	Bagus, saya akan meminta maklum balas anda pada setiap bulan	goal setting	express approval, inform	QAL setting	M-1	modified
MARIA	Penetapan matlamat kami hampir selesai. Biar saya mengesahkan kali kedua dengan anda tentang matlamat ini	goal setting	report	QAL setting	M-1	
{Participant_A}	Ok		answer	QAL setting	Z-1	
MARIA	Tempoh: 29/6/22-29/7/22 Terapi: Beta-Blocker Dos: Bisoprolol 5mg satu kali sehari Peringatan untuk ubatan: 8 pagi Kenalan Kecemasan: Diaktifkan Tinjauan: Mingguan	goal setting	report	Medication details	M-1	modified
{Participant_A}	Ok		answer	medication details	Z-1	
MARIA	Adakah semua ketetapan ini betul?	goal setting	ask	Medication details	M-1	
{Participant_A}	Ya		answer	medication details	Z-1	
MARIA	Bagus! Tetapan sudah siap ðŸ˜Š 😄	goal setting	tell	QAL setting	M-1	
{Participant_A}	Ok terbaik ðŸ		answer	QAL setting	Z-1	
{Participant_A}	Terima kasih		answer	QAL setting	Z-2	
MARIA	Menetapkan matlamat adalah langkah pertama kita untuk berjaya dalam pematuhan ubat. Anda telah melakukannya dengan baik, {Participant_A}! Selain daripada rutin ubatan, jangan lupa untuk mengemas kini rakaman anda di "Report Care App"	goal setting end of protocol	recommend	Medication adherence	M-1	
{Participant_A}	Ok		answer	Medication adherence	Z-1	
MARIA	Selamat pagi {Participant_A}! Tiba masa untuk makan ubat anda 😄	Daily monitoring	remind	Medication reminder	M-1	
{Participant_A}	Ok		answer	Medication reminder	Z-1	
MARIA	Anda boleh mengambil dos pagi anda sekarang. Sila beritahu saya bila anda telah mengambil ubat anda	Daily monitoring	inform	Medication reminder	M-1	modified
{Participant_A}	Sudah diambil tadi pagi		answer	Medication reminder	Z-1	

MARIA	Kerja yang bagus, {Participant_A}! Permulaan yang baik untuk hari ini. Semoga menghadapi hari yang indah dan saya akan kembali kapada anda pagi esok	daily monitoring	applaud	monitoring	M-1	modified
{Participant_A}	Ok, terima kasih		answer	monitoring	Z-1	
MARIA	Selamat pagi {Participant_A} !, Tiba masa untuk makan ubat anda 😄	daily monitoring	remind	Medication reminder	M-1	
MARIA	Anda boleh mengambil dos pagi anda sekarang. Sila beritahu saya bila anda telah mengambil ubat anda	daily monitoring	inform	Medication reminder	M-2	
{Participant_A}	Belum di ambil ubat		answer	medication reminder	Z-1	
MARIA	Berikut adalah jadual ubatan seharian anda: Bisoprolol 5mg satu kali sehari, diambil pada 8 pagi",	daily monitoring	inform	Medication details	M-1	modified
		daily monitoring	inform	medication details	M-2	
MARIA	Anda boleh mengambil dos pagi anda sekarang. Sila beritahu saya bila anda telah mengambil ubat anda	daily monitoring	order	Medication reminder	M-3	modified
{Participant_A}	Sudah diambil ubat		answer	Medication reminder	Z-1	
MARIA	Bagus Saya positif anda boleh berjaya mencapai matlamat satu bulan anda! Saya akan kembali lagi untuk mengingatkan anda pada hari esok	Daily monitoring	applaud, tell	Medication adherence	M-1	
MARIA	Saya baru teringat kita perlu menjawab tinjauan mingguan hari ini. Adakah anda ingin menjawab beberapa soalan berkenaan kualiti hidup anda sejak akhir ini?	Daily monitoring	suggest	QAL	M-1	modified
MARIA	Jawapan anda adalah penting bagi anda dan pakar kardiologi anda untuk memahami pematuhan ubat anda dengan lebih baik	Daily monitoring	inform	QAL	M-2	
{Participant_A}	Ya		answer	QAL	Z-1	
MARIA	Bagus ~ 😀	daily monitoring	applaud	QAL	M-1	
MARIA	Adakah anda mahu melakukan tinjauan ini sekarang?	daily monitoring	ask	QAL	M-2	modified
{Participant_A}	Ya		answer		Z-1	
MARIA	Tinjauan ini adalah berdasarkan cadangan pakar kardiologi dimana yang mengandungi lima soalan pendek untuk membantu anda menilai sendiri gangguan lemah jantung dengan kualiti kehidupan harian anda. Anda perlu menjawab soalan dengan menggunakan nombor 0 hingga 10, sementara:<br><br>"0" bermaksud "tidak mengganggu”<br>"5" bermaksud "Sederhana”<br>"10" bermaksud "Sangat mengganggu”	daily monitoring	inform	QAL	M-1	

MARIA	Soalan 1, dalam tempoh satu minggu yang lalu, adakah sakit lemah jantung ini mengganggu aktiviti am anda?		ask	QAL	M-2	
{Participant_A}	0		answer	QAL	Z-1	
MARIA	Soalan 2, dalam tempoh satu minggu yang lalu, adakah mood anda terganggu oleh sakit lemah jantung ini?		ask	QAL	M-1	
{Participant_A}	5		answer	QAL	Z-1	
MARIA	Soalan 3, dalam tempoh satu minggu yang lalu, adakah sakit lemah jantung mengganggu kerja anda, termasuk kerja-kerja rumah?		ask	QAL	M-1	
{Participant_A}	5		answer	QAL	Z-1	
MARIA	Soalan 4, pada satu minggu yang lalu, adakah anda terganggu oleh sakit lemah jantung semasa berjalan?		ask	QAL	M-1	
{Participant_A}	5		answer	QAL	Z-1	
MARIA	Soalan 5, dalam tempoh satu minggu yang lalu, adakah sakit lemah jantung mengganggu kualiti hidup anda?		ask	QAL	M-1	
{Participant_A}	5		answer	QAL	Z-1	
MARIA	Bagus {Participant_A}. Terima kasih kerana melengkapkan soalan tinjauan ini. ðŸ˜€ 😀	daily monitoring	applaud	QAL	M-1	Modified
MARIA	Jawapan anda akan disimpan untuk rujukan pakar kardiologi anda		inform	QAL	M-2	
{Participant_A}	Ok		answer	QAL	Z-1	
MARIA	Penilaian anda menunjukkan kualiti hidup anda sedang terganggu oleh sakit lemah jantung ini Anda telah menyelesaikan 25% daripada matlamat anda dan anda berada di landasan yang betul untuk mencapai pelan matlamat ubat selama sebulan	daily monitoring	report	QAL	M-1	modified
{Participant_A}	Ok terima kasih		answer		z-1	
MARIA	Semoga menghadapi hari yang indah dan saya akan kembali kepada anda pada pagi esok. â˜€ï¸	daily monitoring	remind	medication adherence	M-1	modified
{Participant_A}	Ok		answer		M-1	
{Participant_A}	MARIA apakah makanan yang saya tidak boleh makan untuk sakit jantung ini?	N/A	query	Diet	z-1	
MARIA	Makanan yang tinggi kandungan garam dan mempunyai kandungan air yang tinggi (contoh: sup) perlu dielakkan. Anda dinasihatkan untuk berjumpa dengan pakar pemakanan untuk mendapatkan pelan pemakanan yang sesuai untuk anda.	N/A	inform	Diet	M-1	
{Participant_A}	Ok		answer	Diet	z-1	
{Participant_A}	Apakah kesan ubat bisoprolol ini bila dimakan setiap hari?		query	Drug side effects	z-1	

MARIA	Bisoprolol boleh menyebabkan degupan jantung menjadi perlahan. Sekiranya kadar degupan jantung anda adalah di bawah 60degupan seminit, sila dapatkan nasihat dari doktor anda	N/A	Inform	Drug side effects	M-1	
{Participant_A}	Ok		answer	Drug side effects	Z-1	

Table 2: Translation of Utterances for Participant A – Original Utterances in Malay Translated into English

Name	Utterance Analysis	Workflow type	Speech act- topic	Topic	Adjacency pair, use number to count how many turns M or Z makes for each topic	Notes/remarks
MARIA	Hi {Participant_A}! \n You can type in your message or record your Utterance voice.\n (If you want to see some examples of the questions you can ask me, write 'help')",	N/A	describe	information on use	M-1	
		N/A				
{Participant_A}	Hi	N/A	answer	information on use	Z-1	
{Participant_A}	What medication should I take today?	N/A	ask	Medication intake	Z-1	
MARIA	Here is the medication plan that your cardiologist has prepared for you.	N/A	answer	medication intake	M-1	modified
MARIA	Log in Patient name : {Participant_A}	N/A		medication intake	M-2	
MARIA	Medication name: Bisoprolol 5mg, once daily	N/A		medication intake	M-3	
MARIA	Would you like to set a goal for medication adherence?	Goal setting	ask	Set-up goal	M-1	
{Participant_A}	Yes, I need to		answer	Set-up goal	Z-1	
MARIA	Setting goals can help you follow your medication routine more effectively. You have one month left before your next appointment. Smiling Face,☺)	Goal setting	explain	medication adherence	M-1	modified. continued from inquiry. typing delay
MARIA	I'm very excited to hear that, {Participant_A}! Let's start our plan towards better quality of life!	Goal setting	express gratitude	medication adherence	M-1	in reply to line 13
{Participant_A}	Ok, thank you, MARIA		accept	medication adherence	Z-1	
MARIA	Medication plan for {Participant_A}: Tablet Bisoprolol 5mg, once daily (8 AM) Duration: 1 month (29/06/2022 - 29/07/2022)	Goal setting	inform	medication plan	M-1	
MARIA	This is the medication plan set by your cardiologist. I'm sure you've been prescribed the necessary medications.	Goal setting	remind	medication plan	M-2	
MARIA	I'm here to help make sure you don't miss your medication. ðŸ™ (Slightly Smiling Face,☺)	Goal setting	tell	medication plan	M-3	
MARIA	Here is a link to an article from the American Heart Association website about the medication you will take. https//www. eart.org/en/health-topics/heart-attack/treatment-of-a-heartattack/cardiac-medications	Goal setting	inform	medication information	M-4	
MARIA	Based on this medication plan, I will set a reminder for you to take your medication at 8 AM.	goal setting	propose	medication setting	M-1	

{Participant_A}	Ok		answer	medication setting	Z-1	
MARIA	Does my setting match your preference? Or would you like to change it with me now? ðŸ˜ (Neutral Face, 😐)	goal setting	ask	medication setting	M-1	
MARIA	You’re also free to adjust the reminder time or increase its frequency before {8 AM}.	goal setting	inform	medication setting	M-2	
{Participant_A}	Ok		answer	medication setting	Z-1	
MARIA	Based on the medication plan, I will set a reminder for you to take your medication at 8 a.m.	N/A	announce	medication timing	M-1	
MARIA	Compliance to your medication is important. It will help improve your health condition and quality of life. As your personal assistant, I will support you every step of the way. ðŸ™‚(Slightly Smiling Face, 🙂),	goal setting	explain	medication adherence	M-1	
{Participant_A}	Ok ðŸ˜Š (ðŸ˜Š Smiling Face With Smiling Eyes)		answer	medication timing	Z-1	
MARIA	Family and friends ðŸ‘¬ ðŸ▯» (▯) can also provide support and confidence to you during your medication adherence journey	goal setting	inform	community support	M-1	not in order
MARIA	Would you like to enable emergency contacts? This is entirely up to you, and I will respect your decision.	goal setting	suggest	contact emergency	M-1	not in order
{Participant_A}	Okay		answer	contact emergency	Z-1	
MARIA	Who would you like to set as an emergency contact? Please provide the name and phone number of the contact you'd like to designate.	goal setting	ask	contact emergency	M-1	modified
MARIA	Who would you like to set as an emergency contact? Please provide the name and phone number of the contact you'd like to designate.		repeated	contact emergency	M-2	repeated
{Participant_A}	{Emergency Contact Name},{Emergency Contact Number}		answer	contact emergency	z-1	
MARIA	Great! I will set it up for you now. Please confirm with a 'Yes.'	goal setting	promise, ask	contact emergency	M-1	modified
{Participant_A}	Yes		answer	contact emergency	z-1	
MARIA	this is your emergency contact: 1. Name: {Emergency Contact Name} 2. Contact Number: {Emergency Contact Number} Please reply with 'Yes' to confirm or 'No' to make changes.	goal setting	report	contact emergency	M-1	modified
{Participant_A}	yes		answer	contact emergency	Z-1	
MARIA	Thank you. Please inform your emergency contact about this setting	goal setting	recommend	contact emergency	M-1	modified
{Participant_A}	ok		answer	contact emergency	Z-1	

MARIA	Would you like to conduct a survey while our program is running? In this survey, I will ask a few simple and general questions to record your feedback on medication adherence. The results of this survey can help you self-assess and provide information to your cardiologist for your next appointment. This survey can be set up weekly or monthly, based on your preference. Would you like to set the survey on a weekly or monthly basis?	goal setting	recommend	QAL	M-1	
{Participant_A}	every week		answer	QAL	Z-1	
MARIA	Great, I will request your feedback every month.	goal setting	express approval, inform	QAL setting	M-1	modified
MARIA	Our goal-setting process is almost complete. Let me confirm these goals with you one more time.	goal setting	report	QAL setting	M-1	
{Participant_A}	ok		answer	QAL setting	Z-1	
MARIA	Duration: 29/6/22 - 29/7/22 Therapy: Beta-Blocker<br>Dosage: Bisoprolol 5mg once daily Medication Reminder: 8 a.m.<br>Emergency Contact:<br>Survey Activated: Weekly	goal setting	report	Medication details	M-1	modified
{Participant_A}	ok		answer	medication details	Z-1	
MARIA	Are all these settings correct?ok	goal setting	ask	Medication details	M-1	
{Participant_A}	yes		answer	medication details	Z-1	
MARIA	Great! The settings are all setðŸ˜Š 😄	goal setting	tell	QAL setting	M-1	
{Participant_A}	Ok great ðŸ		answer	QAL setting	Z-1	
{Participant_A}	Thank you.		answer	QAL setting	Z-2	
MARIA	Setting goals is our first step towards success in medication adherence. You've done a great job, {Participant_A}! Besides your medication routine, don't forget to update your records in the "Report Care App."	goal setting end of protocol	recommend	Medication adherence	M-1	
{Participant_A}	ok		answer	Medication adherence	Z-1	
MARIA	Good morning, {Participant_A}! It's time to take your medication😄	Daily monitoring	remind	Medication reminder	M-1	
{Participant_A}	Ok		answer	Medication reminder	Z-1	
MARIA	You can take your morning dose now. Please let me know once you have taken your medication	Daily monitoring	inform	Medication reminder	M-1	modified
{Participant_A}	Already taken this morning		answer	Medication reminder	Z-1	

MARIA	Good job, {Participant_A}! A great start to the day. Hope you have a wonderful day, and I'll check in with you again tomorrow morning	daily monitoring	applaud	monitoring	M-1	modified
{Participant_A}	Okay, thank you.		answer	monitoring	Z-1	
MARIA	Good morning, {Participant_A}! It's time to take your medication. 😄	daily monitoring	remind	Medication reminder	M-1	
MARIA	You can take your morning dose now. Please let me know once you have taken your medication.	daily monitoring	inform	Medication reminder	M-2	
{Participant_A}	Medication not yet taken.		answer	medication reminder	Z-1	
MARIA	Here is your daily medication schedule: Bisoprolol 5mg once daily, to be taken at 8 a.m.	daily monitoring	inform	Medication details	M-1	modified
		daily monitoring	inform	medication details	M-2	
MARIA	You can take your morning dose now. Please let me know once you have taken your medication.	daily monitoring	order	Medication reminder	M-3	modified
{Participant_A}	Medication has been taken.		answer	Medication reminder	Z-1	
MARIA	Great! I'm believe you can successfully achieve your one-month goal! I'll come back tomorrow to remind you again.	Daily monitoring	applaud, tell	Medication adherence	M-1	
MARIA	I just remembered that we need to complete the weekly survey today. Would you like to answer a few questions about your quality of life recently?	Daily monitoring	suggest	QAL	M-1	modified
MARIA	Your responses are important for you and your cardiologist to better understand your medication adherence.	Daily monitoring	inform	QAL	M-2	
{Participant_A}	Yes		answer	QAL	Z-1	
MARIA	great ~ 😃	daily monitoring	applaud	QAL	M-1	
MARIA	Would you like to complete this survey now?	daily monitoring	ask	QAL	M-2	modified
{Participant_A}	Yes		answer		Z-1	
MARIA	the survey is based on the cardiologist's suggestion [illegible], and contains five short questions to help you self-assess the interference of heart failure with the quality of your daily life. You need answer the questions by using number 0 to 10, while: "0" means "did no interfere" "5" means "interfere moderately" "10" means "interfere completely"	daily monitoring	inform	QAL	M-1	

MARIA	Question 1, in the past one (month), did heart failure interfere your general activity?		ask	QAL	M-2	
{Participant_A}	0		answer	QAL	Z-1	
MARIA	Question 2, in the past one (week), did your mood get interfered by heart failure?		ask	QAL	M-1	
{Participant_A}	5		answer	QAL	Z-1	
MARIA	Question 3, in the past one (week), did heart failure interfere your work, including the housework?		ask	QAL	M-1	
{Participant_A}	5		answer	QAL	Z-1	
MARIA	Question 4, in the past one (week), did you get interfered by heart failure while walking?		ask	QAL	M-1	
{Participant_A}	5		answer	QAL	Z-1	
MARIA	Question 5, in the past one (week), did heart failure interfere your quality of life?		ask	QAL	M-1	
{Participant_A}	5		answer	QAL	Z-1	
MARIA	Great, {Participant_A}. Thank you for completing the survey. ðŸ˜€ 😃	daily monitoring	applaud	QAL	M-1	Modified
MARIA	Your response will be stored for your cardiologist's reference.		inform	QAL	M-2	
{Participant_A}	ok		answer	QAL	Z-1	
MARIA	Your assessment indicates that your quality of life is being affected by heart failure. You have completed 25% of your goal and are on the right track to achieve your one-month medication goal plan.	daily monitoring	report	QAL	M-1	modified
{Participant_A}	ok. Thank You		answer		z-1	
MARIA	Hope you have a wonderful day, and I'll check in with you again tomorrow morning.â˜€ï¸	daily monitoring	remind	medication adherence	M-1	modified
{Participant_A}	Ok		answer		M-1	
{Participant_A}	MARIA apakah makanan yang saya tidak boleh makan untuk sakit jantung ini?	N/A	query	Diet	z-1	
MARIA	Makanan yang tinggi kandungan garam dan mempunyai kandungan air yang tinggi (contoh: sup) perlu dielakkan. Anda dinasihatkan untuk berjumpa dengan pakar pemakanan untuk mendapatkan pelan pemakanan yang sesuai untuk anda.	N/A	inform	Diet	M-1	
{Participant_A}	Ok		answer	Diet	z-1	
{Participant_A}	Apakah kesan ubat bisoprolol ini bila dimakan setiap hari?		query	Drug side effects	z-1	

MARIA	Bisoprolol boleh menyebabkan degupan jantung menjadi perlahan. Sekiranya kadar degupan jantung anda adalah di bawah 60degupan seminit, sila dapatkan nasihat dari doktor anda	N/A	Inform	Drug side effects	M-1	
{Participant_A}	Ok		answer	Drug side effects	Z-1	

Table 3: Utterance analysis for the participant B– Original Utterance is English language with the details of the workflow type, and speech act topic.

Name	Utterance	Workflow type	Speech act- topic	Topic	Adjacency pair, use number to count how many turns M or Z makes for each topic	Notes/remarks
MARIA	Hi {Participant_B}!\n You can type in your message or record your voice.\n (If you want to see some examples of the questions you can ask me, write 'help')	N/A	Describe	Information on used	M-1	
{Participant_B}	How are you ?	N/A	Greeting	send regards	T-1	Not i
MARIA	Hi {Participant_B}, Would you like to set up a goal with me for medication adherence?	Goal setting	ask	setup goal	M-1	
{Participant_B}	Yea		Answer	setup goal	T-1	
MARIA	Setting up a goal can help you keep up on your medication routine more effectively. It's one month up to your next appointment.	Goal Setting	Explain	Medication adherence	M-2	delay for the typing
MARIA	I am so excited to hear this, {Participant_B}. Let's begin our journey towards a good quality of life! ðŸ˜ 😄	Goal Setting	Express gratitude	Medication adherence	M1	
MARIA	Here is a medication plan that your cardiologist has made for you.	Goal setting	inform	Medication plan	M-2	
MARIA	Medication: Bisoprolol 10mg once daily	Goal setting	inform	Medication plan	M-3	
MARIA	Medication time: 8am-12pm	Goal setting	inform	Medication plan	M-4	
MARIA	Period: 30/6/22-30/7/22	Goal setting	inform	Medication plan	M-5	
MARIA	Based on this medication plan, I will set up default reminders to remind your medication at 10am. You are also free to adjust the specific medication time or increase reminder frequency within 8am-12pm and 6-10pm	Goal setting	inform & proprose	Medication setting	M6	
MARIA	Based on this medication plan, I will set up default reminders to remind your medication at 10am. You are also free to adjust the specific medication time or increase reminder frequency within 8am-12pm	Goal setting	inform & proprose	Medication setting	M6	repeated sentence
MARIA	Is my default setting suitable for your preference? Or would you like to adjust it with me now? ðŸ˜ 😄	goal setting	ask	medication setting	M7	
{Participant_B}	Yes		answer	Medication setting	T1	
MARIA	Great! ðŸ˜€ 😀	Goal setting	Express gratitude	Medication setting	M1	
ARIA	Keeping up with your medication is important. It will help you improve your health condition and quality of life. As your personal assistant, I will assist you all the way. ðŸ™ 🙂	Goal setting	express support	Medication adherence		
MARIA	Family and friends ðŸ‘¬ðŸ » ? could also give us important support and confidence during our journey of medication adherence	goal setting	inform	community support	M3	

MARIA	Would you like to activate this emergency contact person? This is entirely up to you, I will follow your decision.	goal setting	recommend	contact emergency	M4	
{Participant_B}	ok		answer	contact emergency	T1	
MARIA	Cool! I will activate this emergency contact person for you.	goal setting	express gratitude & inform	contact emergency	M1	
MARIA	Please state your emergency contact person's : 1. Full Name: 2. Contact Number:	goal setting	ask	contact emergency	M2	
{Participant_B}	{Emergency Contact Name}		answer	contact emergency	T1	
{Participant_B}	{Emergency Contact Number}		answer	contact emergency	T2	
MARIA	Excellent! I will set this up for you now.	goal setting	express my admiration & report	contact emergency	M1	
MARIA	This is your emergency contact person: 1. Name:{Emergency Contact Name}. Contact Number: {Emergency Contact Number} Please reply 'Yes' to confirm or 'No' to edit.	goal setting	report	contact emergency	M2	
{Participant_B}	Yes		answer	contact emergency	T1	
MARIA	Thank you. Your emergency contact person has now been set.	goal setting	report	contact emergency	M1	
MARIA	Please remember to inform your emergency contact person of this arrangement.	goal setting	report	contact emergency	M2	
MARIA	Would you also like to do some surveys during our journey? ðŸ˜€ 😀	goal setting	recommend	QAL setting	M3	
MARIA	In the survey, I will ask some simple and general questions to record your feedback about medication adherence.	goal setting	Explain	QAL Setting	M4	
{Participant_B}	ok		answer	QAL Setting	T1	
MARIA	These survey outcomes could help you self evaluate yourself and provide information to cardiologist for your next appointment	goal setting	Explain	QAL Setting	M1	Typing delay followed by line 31
MARIA	This survey could be set to either weekly or monthly, based on your preference. Would you like to select weekly or monthly surveys?	goal setting	recommend	QAL Setting	M2	Typing delay followed by line 31
{Participant_B}	Weekly		answer	QAL Setting	T1	
MARIA	Excellent, I will request for your feedback, weekly	goal setting	1. Express approval 2. Inform	contact emergency	M1	
MARIA	Our goal setting is nearly done. Just let me double confirm with you about our goal.	goal setting	report	QAL setting	M2	

MARIA	Period: 30/6/22- 30/7/22 Therapy: Beta-Blocker Dose: Bisoprolol 10mg once daily Medication Reminder: 10am Emergency Contact: Activated Survey: Weekly	goal setting	report	1. Goal setting details<br>2. Emergency contact details	M3	
MARIA	Would you like to confirm if these are all good to you? ðŸ˜€ 😀	goal setting	ask	1. Goal setting details<br>2. Emergency contact details	M4	
{Participant_B}	Yes		Answer	1. Goal setting details<br>2. Emergency contact details	T1	
MARIA	Great! All done! ðŸ˜ 😄		Express approval	1. Goal setting details<br>2. Emergency contact details	M1	
MARIA	Setting up the goal is the first step to our success of medication adherence. You've done well, {Participant_B}! Also, besides the medication routine, don't forget to keep up on your recordings in the Report Care App	goal setting	Inform	Medication adherence	M2	COmpletion of goal setting
MARIA	Good Morning, {Participant_B}! It's time to take your medication ðŸ˜ 😄	Daily monitoring	inform	Medication Reminder	M1	Day 2
MARIA	Here is your full day medication schedule: Bisoprolol 10mg once daily, taken at 8am "	Daily monitoring	inform	1. Medication Reminder<br>2. Medication deteails	M2	
MARIA	You can take your morning dose now. Let me know when you have successfully taken your medication. ðŸ™ 🙂	Daily monitoring	order	Medication Reminder	M3	
{Participant_B}	Done		answer	Medication Reminder	T1	
MARIA	Good job, {Participant_B}! What a good start to the day. Have a lovely day and I will come back to you tomorrow morning.	Daily monitoring	Applaud	Medication Reminder	M1	
MARIA	Good Morning, {Participant_B}! It's time to take your medication ðŸ˜ 😄	Daily monitoring	Inform	Medication Reminder	M1	Day 3
MARIA	Here is your full day medication schedule: Bisoprolol 10mg once daily, taken at 8am	Daily monitoring	inform	1. Medication Reminder<br>2. Medication deteails	M2	
MARIA	You can take your morning dose now. Let me know when you have successfully taken your medication. ðŸ™,🙂	Daily monitoring	order	Medication Reminder	M3	
{Participant_B}	No I forgot		answer	Medication Reminder	T1	skip a step
MARIA	Here is your full day medication schedule: Bisoprolol 10mg once daily, taken at 8am	Daily monitoring	inform	Medication Reminder	M!	
MARIA	Is it because of our reminder set-up?	Daily monitoring	ask	Medication reminder setting	M2	
{Participant_B}	No		Answer	Medication reminder setting	T1	

MARIA	You are still within the time frame to take your morning dose. You may take your morning dose now.	Daily monitoring	Inform	Medication Reminder	M1	
MARIA	Let me know when you finish your medication ðŸ™☺	Daily monitoring	Order	Medication Reminder	M2	
{Participant_B}	ok		Answer	Medication reminder	T1	
{Participant_B}	Done		Answer	Medication reminder	T2	modified, did not continue for teh next step
MARIA	Excellent. I'm positive you can successfully achieve your one month goal!	Daily monitoring	applaud, tell	Medication adherence	M1	
MARIA	I just remembered we also need to the weekly survey today Would you like to answer some questions about the quality of your life of late?	Daily monitoring	suggest	QAL	M2	
MARIA	Your answers will be important for you and your cardiologist to understand your medication adherence better	Daily monitoring	inform	QAL	M3	
{Participant_B}	Yes		answer	QAL	T1	
MARIA	Self- evaluation is important for both yourself and the cardiologist to better understand your current medication routine	Daily monitoring	Inform	QAL	M1	modified
MARIA	The survey is based on the cardiologist's suggestion ðŸ‘¨â€ âš•ï ¸ and contains five short questions to help you self-assess the interference of heart failure with the quality of your daily life.<br><br>You need answer the questions by using number 0 to 10, while:<br><br>"0" means "did no interfere"<br>"5" means "interfere moderately"<br>"10" means "interfere completely"	Daily monitoring	Inform	QAL	M2	
MARIA	Question 1, in the past one (week), did heart failure interfere your general activity?	Daily monitoring	ask	QAL	M3	
{Participant_B}	0	Daily monitoring	answer	QAL	T1	
MARIA	Question 2, in the past one (week), did your mood get interfered by heart failure?	Daily monitoring	ask	QAL	M1	
{Participant_B}	0	Daily monitoring	answer	QAL	T1	
MARIA	Question 3, in the past one (week), did heart failure interfere your work, including the housework?	Daily monitoring	ask	QAL	M1	
{Participant_B}	0	Daily monitoring	answer	QAL	T2	

MARIA	Question 4, in the past one (week), did you get interfered by heart failure while walking?	Daily monitoring	ask	QAL	M1	
{Participant_B}	0	Daily monitoring	answer	QAL	T2	
MARIA	Question 5, in the past one (week), did heart failure interfere your quality of life?	Daily monitoring	ask	QAL	M1	
{Participant_B}	0	Daily monitoring	answer	QAL	T2	
MARIA	Great, {Participant_B}. Thank you for completing the survey. ðŸ˜€ 😃 Your response will be stored for your cardiologist's reference	Daily monitoring	applaud, tell	QAL	M1	
MARIA	Your assessment shows the quality of your life is not interfered by heart failure. You have completed 25% of your goal and you are on track to achieve the one-month medication goal	Daily monitoring	Report	QAL	M2	
MARIA	I will be back tomorrow morning for your daily reminder.	Daily monitoring	Inform	Medication reminder	M1	

Table 4: Utterance analysis for the participant C– Original Utterance is English language with the details of the workflow type, and speech act topic.

Name	Utterance	Workflow type	Speech act- topic	Topic	Adjacency pair, use number to count how many turns M or Z makes for each topic	Notes/remarks
MARIA	Hi {Participant_C}!\n You can type in your message or record your voice.\n (If you want to see some examples of the questions you can ask me, write 'help'	N/A	Describe	Information on used	M-1	
{Participant_C}	Hi	N/A	Greeting	send regards	C-1	
MARIA	Hi {Participant_C}! Would you like to set up a goal with me for medication adherence	Goal setting	ask	setup goal	M-1	delay for the typing
MARIA	Setting up a goal can help you keep up on your medication routine more effectively. It's one month up to your next appointment! ðŸ˜€ 😀	Goal Setting	Explain	Medication adherence	M-2	
{Participant_C}	Yes please		Answer	setup goal	C-1	
MARIA	I am so excited to hear this, {Participant_C}. Let's begin our journey towards a good quality of life! ðŸ˜ 😄	Goal Setting	Express gratitude	Medication adherence	M1	
MARIA	Here is a medication plan that your cardiologist has made for you.	Goal setting	inform	Medication plan	M-2	
MARIA	Name: {Participant_C}, Bisoprolol 1.25mg, Once a day.	Goal setting	inform	Medication plan	M-3	
{Participant_C}	ok, tq	Goal setting	Answer	setup goal	C1	
MARIA	keeping up with your medication is important. It will help you improve your health condition and quality of life. As your personal assistant, I will assist you all the way. ðŸ™ 🙂	Goal setting	Inform support	Medication	M1	
MARIA	Family and friends ðŸ'¬ðŸ » ⍰ could also give us important support and confidence during our journey of medication adherence	Goal setting	inform	community support	M2	
MARIA	Would you like to activate this emergency contact person? This is entirely up to you, I will follow your decision.	Goal setting	recommend	contact emergency	M3	
{Participant_C}	Emergency contact person 1 {Emergency Contact Name}	Goal setting	answer	contact emergency	C1	
{Participant_C}	{Emergency Contact Number}	Goal setting	answer	contact emergency	C2	
MARIA	Cool! I will activate this emergency contact person for you.	Goal setting	express gratitude & inform	contact emergency	M1	
MARIA	Emergency contact person's : 1. Full Name:{Emergency Contact Name}. Contact Number: {Emergency Contact Number}	Goal setting	report	contact emergency	M2	
MARIA	reply 'Yes' to confirm or 'No' to edit.	Goal setting	Ask	contact emergency	M3	
{Participant_C}	Yes	Goal setting	answer	contact emergency	C1	

MARIA	Thank you. Your emergency contact person has now been set.	Goal setting	report	contact emergency	M1	
MARIA	Please remember to inform your emergency contact person of this arrangement.	Goal setting	Inform	contact emergency	M2	
MARIA	Would you also like to do some surveys during our journey? ðŸ˜€ 😀	Goal setting	recommend	QAL setting	M3	
{Participant_C}	ok	Goal setting	answer	QAL Setting	C1	
MARIA	In the survey, I will ask some simple and general questions to record your feedback about medication adherence	Goal setting	Explain	QAL Setting	M4	delay for the typing follow by the line 22
{Participant_C}	ok	Goal setting	answer	QAL Setting	C1	
MARIA	These survey outcomes could help you self evaluate yourself and provide information to cardiologist for your next appointment	Goal setting	Explaine	QAL Setting	M1	
MARIA	This survey could be set to either weekly or monthly, based on your preference. Would you like to select weekly or monthly surveys?	Goal setting	Ask	QAL setting	M2	
{Participant_C}	weekly	Goal setting	Answer	QAL setting	C1	
MARIA	Excellent, I will request for your feedback, weekly.	Goal setting	1. Express approval 2. Inform	QAL setting	M1	
{Participant_C}	tq	Goal setting	answer	expression	C1	
MARIA	Our goal setting is nearly done. Just let me double confirm with you about our goal.	Goal setting	expression	QAL setting	M1	
MARIA	Period: 01/07/2022- 31/07/2022 Dose: Bisoprolol, 1.25mg once a day. Emergency Contact: Activated Survey: weekly	Goal setting	Inofrmation	medication addhrence	M2	
MARIA	Would you like to confirm if these are all good to you? ðŸ˜€ 😀	Goal setting	ask	medication	M3	
{Participant_C}	ok tq	Goal setting	answer	Medication adherence	C1	
MARIA	Great! All done! ðŸ 😄	Goal setting	Express approval	1. Goal setting details 2. Emergency contact details	M1	
MARIA	Setting up the goal is the first step to our success of medication adherence. You've done well, {Participant_C}!	Goal setting	express & Inform	Medication adherence	M2	
{Participant_C}	tq	Goal setting	answer	Medication adherence	C1	
MARIA	Also, besides the medication routine, don't forget to keep up on your recordings in the Report Care App	Daily monitoring	inform	1. Medication Reminder 2. Medication deteails	M1	
{Participant_C}	ok		answer	Medication adherence	C1	

MARIA	Good Morning, {Participant_C}! It's time to take your medication ðŸ˜ 😄	Daily monitoring	inform	Medication Reminder	M1	
{Participant_C}	ok will d	Daily monitoring	answer	Medication Reminder	C1	
MARIA	Here is your full day medication schedule: Bisoprolol, 1.25mg once a day, taken at 8am.	Daily monitoring	remind	Medication adherence	M1	
MARIA	You can take your morning dose now. Let me know when you have successfully taken your medication. ðŸ™ 🙂	Daily monitoring	remind	Medication adherence	M2	
{Participant_C}	ok 8 am to fake medicine		answer	Medication adherence	C1	
{Participant_C}	Taken		answer	Medication adherence	C2	
MARIA	Good job, {Participant_C}! What a good start to the day. Have a lovely day and I will come back to you tomorrow morning. ðŸ™ 🙂	Daily monitoring	applaud	Medication adherence	M1	
MARIA	Good Morning, {Participant_C}! It's time to take your medication ðŸ˜ 😄	Daily monitoring	Greeting, remind	Medication Reminder	M1	Day 2
{Participant_C}	Hi {Participant_C}!\n You can type in your message or record your voice.\n (If you want to see some examples of the questions you can ask me, write 'help')					Patient log out
{Participant_C}	hi		Greeting		C1	Day 2
MARIA	Good Morning, {Participant_C}! â˜€ï¸ â˜€ï¸ â˜€ï¸ â˜€ï¸ It's time to take your medication .ðŸ˜ 😄	Daily monitoring	remind	Medication Reminder	M1	
{Participant_C}	ok		Answer	Medication	C1	
MARIA	Here is your full day medication schedule: Bisoprolol, 1.25mg once a day, taken at 8am.	Daily monitoring	remind	medication adherence	M1	
{Participant_C}	ok		answer	medcation	C1	
MARIA	You can take your morning dose now. Let me know when you have successfully taken your medication. ðŸ™ 🙂	Daily monitoring	remind	medication adherence	M1	
{Participant_C}	taken my medicine		answer	Medication adherence	C1	
MARIA	Excellent.	Daily monitoring	applaud	Medication adherence	M1	
MARIA	I'm positive you can successfully achieve your one month goal.	Daily monitoring	applaud	Medication adherence	M2	
{Participant_C}	tq		Answer	Medication adherence	C1	
MARIA	Good Morning, {Participant_C}! Rise and shine. It's time to take your medication ðŸ˜ 🙂	Daily monitoring	Greeting, remind	Medication adherence	M1	Day 3 forgot medication
{Participant_C}	i forgot to take my medicine for today		Answer	Medication adherence	C1	

MARIA	Dont worry, you still can take your medication now.	Daily monitoring	Explain and remind	Medication adherence	M1	Modified
MARIA	Dont worry, you are still within the time frame to take your morning dose.	Daily monitoring	Explain and remind	Medication adherence	M2	Modified
{Participant_C}	ok	Daily monitoring	Answer	Medication adherence	C1	
MARIA	You may take your morning dose now.	Daily monitoring	remind	Medication adherence	M1	
MARIA	ðŸ˜† 😆	Daily monitoring	emoticon, expression	Medication adherence	M2	
{Participant_C}	tq for info	Daily monitoring	1. answer<br>2. appreciation	Medication adherence	C1	
MARIA	You forgot to take your medication. Is it because of our reminder set up?	Daily monitoring	ask	Medication adherence	M1	
{Participant_C}	simply i forgot to take because too busy	Daily monitoring	explain	Medication	C1	
MARIA	Oh, i see. it's okay. ðŸ˜€ 😀	Daily Monitoring	emoticon, expression	Medication adherence	M1	
MARIA	I just remembered we also need to the weekly survey today	Daily Monitoring	Suggest	QAL	M2	
{Participant_C}	ok	Daily monitoring	answer	QAL	C1	
MARIA	Would you like to answer some questions about the quality of your life?	Daily Monitoring	Suggest	QAL	M1	
MARIA	Your answers will be important for you and your cardiologist to understand your medication adherence better.	Daily monitoring	inform	QAL	M2	
{Participant_C}	Yes	Daily Monitoring	Answer	QAL	C1	
MARIA	Cool~ ðŸ˜ƒ 😃	Daily monitoring	emoticon, expression	QAL	M1	
MARIA	the survey is based on the cardiologist's suggestion ¸ and contains five short questions to help you self-assess the interference of heart failure with the quality of your daily life. You need answer the questions by using number 0 to 10, while:<br><br>"0" means "did no interfere"<br>"5" means "interfere moderately"<br>"10" means "interfere completely"	Daily monitoring	inform	QAL	M2	
{Participant_C}	ok	Daily monitoring	answer	QAL	C1	

{Participant_C}	ok	Daily monitoring	answer	QAL	C2	repeat
MARIA	Question 1, in the past one (month), did heart failure interfere your general activity?	Daily monitoring	ask	QAL	M1	
{Participant_C}	5	Daily monitoring	answer	QAL	C1	
MARIA	Question 2, in the past one (week), did your mood get interfered by heart failure?	Daily monitoring	ask	QAL	M1	
{Participant_C}	0	Daily monitoring	answer	QAL	C1	
MARIA	Question 3, in the past one (week), did heart failure interfere your work, including the housework?	Daily monitoring	ask	QAL	M1	
{Participant_C}	5	Daily monitoring	answer	QAL	C1	
MARIA	Question 4, in the past one (week), did you get interfered by heart failure while walking?	Daily monitoring	ask	QAL	M1	
{Participant_C}	5	Daily monitoring	answer	QAL	C1	
MARIA	Question 5, in the past one (week), did heart failure interfere your quality of life?	Daily monitoring	ask	QAL	M1	
{Participant_C}	5	Daily monitoring	answer	QAL	C1	
MARIA	Great, {Participant_C}. Thank you for completing the survey. ðŸ˜€ 😀	Daily monitoring	applaud, tell	QAL	M1	
MRIA	Your response will be stored for your cardiologist's reference.	Daily monitoring	report	QAL	M2	
{Participant_C}	welcome	Daily Monitorng	answer	QAL	C1	
MARIA	I will be back for your medication reminder tomorrow morning at 8 am. Have a lovely day.ðŸ¥°ðŸ¥° 🥰🥰	Daily Monitorng	Inform	Medication adherence	M1	
{Participant_C}	ok	Daily Monitorng	answer	Medication ahderence	C1	
{Participant_C}	if my feet swollen... iks it because lf water retention in my body	symptoms complaint	Ask	Disease information	C1	Extra question
MARIA	HI {Participant_C}, based on your medical history record you have heart failure. This might be one of the symptoms you are experiencing. If you develop leg swelling, please follow your doctors advice with 500cc fluid intake per day.	symptoms complaint	Explained	Disease information	M1	Extra question
MARIA	if you having the problem, please seek for medical attention if your symptoms are worsening	symptoms complaint	Inform, advice	Disease information	M2	Extra question

{Participant_C}	breathing difficulty after walking a few steps	symptoms complaint	Ask	Disease information	C1	Extra question
MARIA	Difficulty of breathing is one of the common symptom in heart failure patient. if you have difficulty of breathing and worsening, please seek for medical attention immediately.	symptoms complaint	Explained, advice	Disease information	M1	Extra question
{Participant_C}	tq	symptoms complaint	appreciation	Disease information	C1	Extra question
MARIA	Please control your fluid intake with 500cc per day, low salt diet, and compliance to your meidication	daily monitoring	reminder	Daily monitoring	M1	Extra question
{Participant_C}	ok	daily monitoring	appreciation	Daily monitoring	C1	Extra question

Table 5: Translation Excerpt from the coding analysis: Translation utterance / Conversation from Malay language to English language

Name	Utterance/ Conversation*	Workflow type	Speech act-topic	Topic	Adjacency pair, use a number to count how many turns M or Z makes for each topic	Notes/Remarks
MARIA	Hi Zub! \n You can type in your message or record your voice.\n (If you want to see some examples of the questions you can ask me, write 'help')",	N/A	describe	information on use	M-1	
Participant 1	Hi	N/A	answer	information on use	Z-1	
Participant 1	What medication should I take today?	N/A	ask	Medication intake	Z-1	
MARIA	Here is the medication plan that your cardiologist has prepared for you.	N/A	answer	medication intake	M-1	modified
	Log in Patient's name : participant 1	N/A		medication intake	M-2	
	Medication name: Bisoprolol 5mg, once daily	N/A		medication intake	M-3	
	Would you like to set a goal for medication adherence?	Goal setting	ask	Set-up goal	M-1	
Participant 1	Yes, I need to		answer	Set-up goal	Z-1	
MARIA	Setting goals can help you follow your medication routine more effectively. You have one month left before your next appointment. Smiling Face, ☺)	Goal setting	explain	medication adherence	M-1	modified. continued from inquiry. typing delay
	I'm very excited to hear that, Participant_1 Let's start our plan towards better quality of life!	Goal setting	express gratitude	medication adherence	M-1	in reply to line 13
Participant 1	Ok, thank you, MARIA		accept	medication adherence	Z-1	
MARIA	Medication plan for {Participant 1: Tablet Bisoprolol 5mg, once daily (8 AM) Duration: 1 month (29/06/2022 - 29/07/2022)	Goal setting	inform	medication plan	M-1	

	This is the medication plan set by your cardiologist. I’m sure you’ve been prescribed the necessary medications.	Goal setting	remind	medication plan	M-2	
	I’m here to help make sure you don’t miss your medication. ðŸ™ (Slightly Smiling Face, 🙂)	Goal setting	tell	medication plan	M-3	
	Here is a link to an article from the American Heart Association website about the medication you will take. https//www. eart.org/en/health-topics/heart-attack/treatment-of-a-heartattack/cardiac-medications	Goal setting	inform	medication information	M-4	
	Based on this medication plan, I will set a reminder for you to take your medication at 8 AM.	goal setting	propose	medication setting	M-1	
Participant 1	Ok		answer	medication setting	Z-1	
