# Supplementary material for: MARIA (Medical Assistance and Rehabilitation Intelligent Agent) for Medication Adherence in Patients With Heart Failure: Empirical Results From a Wizard of Oz Systematic Conversational Agent Design Clinical Protocol
Source: JMIR Cardio. 2025 Apr 10;9:e55846. doi: 10.2196/55846 (PMC12022519; doi:10.2196/55846)
Supplement: Multimedia Appendix 3 [file cardio_v9i1e55846_app3.docx]

| Name | Utterance/conversation^a^ | Workflow type | Speech act: topic | Topic | Adjacency pair, use a number to count how many turns M or Z makes for each topic | Notes/remarks |
| --- | --- | --- | --- | --- | --- | --- |
| MARIA^b^ | Hi Zub! \n You can type in your message or record your voice.\n (If you w  ant to see some examples of the questions you can ask me, write 'help')", | N/A^c^ | Describe | Information on use | M-1 | N/A |
|  |  |  |  |  |  |  |
| Participant 1 | Hai | N/A | Answer | Information on use | Z-1 | N/A |
| Participant 1 | Apa ubat yang perlu saya ambil hari ini? | N/A | Ask | Medication intake | Z-1 | N/A |
| MARIA | Berikut Hialeah pelan ubat yang telah dibuat Oleh pakar kardiologi anda untuk anda | N/A | Answer | Medication intake | M-1 | Modified |
|  | Log in Patient’s name : participant 1 | N/A |  | Medication intake | M-2 | N/A |
|  | Nama ubat: Bisoprolol 5mg, satu kali sehari" | N/A |  | Medication intake | M-3 | N/A |
|  | Adakah anda ingin menetapkan matlamat untuk pematuhan ubat? | Goal setting | Ask | Setup goal | M-1 | N/A |
| Participant 1 | Ya perlu |  | Answer | Setup goal | Z-1 | N/A |
| MARIA | Dengan menetapkan matlamat, boleh membantu anda menjalani rutin pengambilan ubat anda dengan lebih berkesan. Anda mempunyai sebulan lagi sebelum temujanji anda yang seterusnya! ðŸ™ (Slightly Smiling Face‚🙂) | Goal setting | Explain | Medication adherence | M-1 | Modified; continued from the inquiry; typing delay |
|  | Saya sangat teruja mendengarnya, participant 1! Mari mulakan pelan kita ke arah kualiti hidup yang lebih baik! | Goal setting | Express gratitude | Medication adherence | M-1 | In reply to line 13 |
| Participant 1 | Ok terima kasih maria |  | Accept | Medication adherence | Z-1 | N/A |
| MARIA | Pelan pengambilan Ubat untuk for Zub Tablet Bisoprolol 5mg satu kali sehari (8 pagi) Tempoh: 1bulan (29/06/2022- 29/07/2022) | Goal setting | Inform | Medication plan | M-1 | N/A |
|  | Ini ialah pelan ubat yang telah ditetapkan oleh pakar kardiologi anda untuk anda. Saya pasti bahawa anda telah diberikan ubat-ubatan yang anda perlukan | Goal setting | Remind | Medication plan | M-2 | N/A |
|  | Saya di sini untuk membantu dalam memastikan anda tidak terlepas pengambilan ubat anda ðŸ™ (Slightly Smiling Face‚🙂) | Goal setting | Tell | Medication plan | M-3 | N/A |
|  | Ini adalah pautan untuk artikel dari laman web Persatuan Jantung Amerika mengenai maklumat ubat yang anda akan ambil. https//www.heart.org/en/health-topics/heart-attack/treatment-of-a-heart-attack/cardiac-medications | Goal setting | Inform | Medication information | M-4 | N/A |
|  | Berdasarkan pelan ubat ini, saya akan menetapkan peringatan untuk mengingatkan pengambilan ubat anda pada 8 pagi | goal setting | Propose | Medication setting | M-1 | N/A |
| Participant 1 | Ok |  | Answer | Medication setting | Z-1 | N/A |

^a^Participant 1 original conversation or utterance is in the Malay language, and the English translation is attached in Multimedia Appendix 2: Table 5

^b^MARIA: Medical Assistance and Rehabilitation Intelligent Agent.

^c^N/A: not applicable.
